# Insight into human pubertal growth by applying the QEPS growth model

**DOI:** 10.1186/s12887-017-0857-1

**Published:** 2017-04-19

**Authors:** Anton Holmgren, Aimon Niklasson, Lars Gelander, A. Stefan Aronson, Andreas F.M. Nierop, Kerstin Albertsson-Wikland

**Affiliations:** 1 Göteborg Pediatric Growth Research Center, Department of Pediatrics, Institute of Clinical Sciences, Sahlgrenska Academy at University of Gothenburg, SE-41685 Gothenburg, Sweden; 2 Hallands Hospital Halmstad, Halmstad, Sweden; 3 Muvara bv, Multivariate Analysis of Research Data, Leiderdorp, The Netherlands; 4 Department of Physiology/Endocrinology, Institute of Neuroscience and Physiology, The Sahlgrenska Academy at University of Gothenburg, SE-40530 Gothenburg, Sweden

**Keywords:** Puberty, Growth model, Onset of puberty, Peak height velocity, End of puberty, Duration of puberty, Data quality, Cumulative distribution, Confidence interval

## Abstract

**Background:**

Computerized mathematical models describing absolute and relative individual growth during puberty in both cm and standard deviation (SD)-scores are lacking. The present study aimed to fill this gap, by applying the QEPS-model that delineates mathematically the specific pubertal functions of the total growth curve.

**Methods:**

Study population used was the individual growth curves of the longitudinally followed cohort GrowUp1974 Gothenburg (*n* = 2280). The QEPS-model describes total height as *(T)otal-*function*:* a combination of four shape-invariant growth functions, modified by time-scale and height-scale parameters: a (*Q)uadratic-*function for the continuous growth from fetal life to adulthood; a negative *(E)xponential-*function adds the rapid, declining fetal/infancy growth; a *(P)ubertal-*function the specific pubertal growth spurt; a *(S)top-*function the declining growth until adult height. A constructed variable, *MathSelect*, was developed for assessing data-quality. CIs and SD-scores for growth estimates were calculated for each individual.

QEPS-model estimates used for pubertal growth; from the *T-*function: onset of puberty as minimal height velocity (*AgeT*_*ONSET*_); mid-puberty as peak height velocity (*AgeT*_*PHV*_); end of puberty as height velocity decreased to 1 cm/year (*AgeT*_*END*_); duration of different intervals and gain (*AgeT*_*ONSET–END*_ and *Tpubgain*); from the *P-*function: onset of puberty, estimated as growth at 1% or 5% (*AgeP1*_*,*_ *AgeP5)*; mid-puberty as 50% (*AgeP50*) and PHV (*AgeP*_*PHV*_); end of pubertal growth at 95 or 99% (*AgeP95, AgeP99)*; duration of different intervals and pubertal gain (*Ppubgain; P*_*max*_); from the *QES-*function: gain (*QESpubgain)*_*.*_

**Results:**

Application of these mathematical estimates for onset, middle and end of puberty of *P-*function*, QES-*function, and *T-*function during puberty showed: the later the onset of puberty, the greater the adult height; pubertal gain due to the *P*-function growth was independent of age at onset of puberty; boys had higher total gain during puberty due to *P-*function growth than to *QES*-function growth; for girls it was reversed.

**Conclusions:**

QEPS is the first growth model to provide individualized estimates of both the specific pubertal growth function and the total growth during puberty, with accompanying SD-scores and Cis for each individual. These QEPS-derived estimates enable more in-depth analysis of different aspects of pubertal growth than previously possible.

**Electronic supplementary material:**

The online version of this article (doi:10.1186/s12887-017-0857-1) contains supplementary material, which is available to authorized users.

## Background

Pubertal growth is unique to humans [1]. For the individual, puberty constitutes a dramatic change in both the magnitude and tempo of growth. In a healthy population, there is wide variation in when children enter puberty, both within and between genders [2, 3]. Thus, accurately describing this period of growth is challenging due to the complexity of the changes that occur and the differences observed between individuals. At present, methods for modelling pubertal growth are limited, and no existing growth references allow appropriate adjustments for the onset of puberty. Furthermore, variations between individuals add to the challenges of modeling growth, particularly when they are considered to be related to maturation (biological age) rather than to chronological age. The pattern of pubertal growth has also changed over time, and varies between different populations [4, 5]. The large variations in both the timing of puberty and amount of growth which are apparent among individuals and between populations highlight the need for individualized equations and estimates describing pubertal growth.

The years preceding puberty are characterized by a period of slowly declining height velocity [3, 6]. The *onset of pubertal growth* can be identified based on the smallest height velocity that precedes what has been referred to as the take-off, onset, nadir or insertion point [2, 3, 7]. In previous studies it has also been described as the point at the beginning of the pubertal growth spurt where height increased by 0.3 standard deviation (SD) scores, or as the point 2 years before peak height velocity (PHV) [6, 8, 9]. Thereafter, height velocity rapidly increases, and the SD of both observed height and height velocity for any population increases due to the broad variation in the timing of puberty [3]. *PHV* – the mid-point in puberty where growth is most pronounced – has often been used as the only estimate of pubertal growth in previous research. The easiest, and probably most unreliable, way of defining age at PHV is by estimating the age at which height increases most from the growth curve, either by visual inspection or using a specific puberty ruler [10]. Age at PHV in contemporary research may also be defined by visual inspection of the change in growth velocity on a computer-generated height velocity chart [2, 11]. Another way of defining age at PHV is to take the age at the midpoint in the interval between the two height measurements with the greatest calculated yearly height increment [6, 12]. The latter is reliable when height measurements are available every 3 months, but less precise when measurements are taken at longer intervals; there is a risk of under-estimating the age at PHV when measurements are at 6- or 12-month intervals [9, 13]. The *end of pubertal growth* has typically not been specifically identified, and measurements have instead been based on when adult height was attained. Therefore, the total pubertal height gain has been defined as the amount of growth observed from the onset of pubertal growth until adult height, with the duration of pubertal growth defined as the time period from the onset of puberty to the attainment of adult height [2, 12, 14].

Few studies have attempted to describe the whole pattern of pubertal growth in a detailed manner, including separate estimations of growth for the onset, middle and end of the pubertal period. In 1980, Taranger & Hägg described a way to estimate the duration and gain of pubertal growth based on visual inspection of individual growth charts [15]. Mathematical models have also been used to describe growth from birth to adult height [16–19]. The ICP-model (Infancy-Childhood-Puberty), developed in Gothenburg by Karlberg et al. was the first model to use three different mathematical functions related to the periods of biological growth [12, 20, 21]. Thus, during the pubertal years, total growth can be separated into the childhood component and the pubertal component. However, the pubertal component of the ICP-model has a fixed form, such that only the timing of pubertal growth, not the magnitude of the specific pubertal growth function, can be individualized. This means that the model assumes that all variations in pubertal growth in individuals of the same gender are related to differences in the childhood growth component that is still ongoing during the pubertal period [21]. The first published growth model that allowed for individualization of the pubertal growth was the SITAR-model by Cole et al. [22]. The model generates a growth curve and three subject-specific parameters (size, tempo and velocity) that can be adjusted to describe individual growth patterns. However, this model cannot separate growth during puberty into different components, instead providing only one mean shape-invariant growth function.

**Fig. 1:**
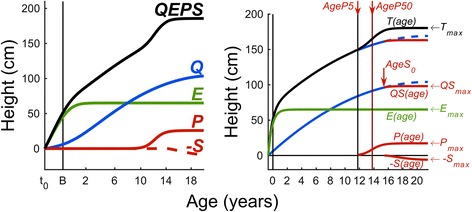
The QEPS-model. *Left panel*: The total height (*QEPS*) is the sum of four growth functions: a quadratic growth function (*Q*), a negative exponential growth function (*E*), a pubertal growth function (*P*) and a stop function (*S*) modelling the end of growth for function *Q*. B = birth, t_0_ = about 6 weeks after conception. Birth is marked with a vertical line. Age scale below 3 years is stretched out. *Right panel*: QEPS model for total height, *T(age),* = *E(age)* + *QS(age)* + *P(age)*, with *QS(age)* = *Q(age)* – *S(age)*. *T*_*max*_ *= E*_*max*_ + *QS*_*max*_ *+ P*_*max*_. *AgeP5* and *AgeP50* mark the ages where 5% and 50% of *P*_*max*_ are reached, *AgeS*_*0*_ marks the age where the *S*-function is starting. Originally published in Journal of theoretical biology 2016;406:143–65, Nierop AF et al., used by permission of Elsevier journals

The first model to describe individual longitudinal human growth and its different phases from fetal life until adult height is the QEPS-model by Nierop et al. [23]. The model was constructed with a combination of four distinct shape-invariant growth functions: Quadratic (*Q*), Exponential (*E*), Pubertal (*P*) and Stop (*S*), (Fig. 1). All four functions have an individual height-scale parameter, and the E- and P-functions also have individual time-scale parameters; giving six modifying parameters in total to describe individual growth. Basic features of the *Q* and *E* functions have been used in previous prediction models [24–26].

In the present study we implement novel estimates of pubertal growth from the QEPS mathematical growth model in cm and SD-scores both at the individual and the group level. The model calculates both the specific pubertal *P-*function during puberty and the from prepubertal period ongoing *QES-*functions*,* as well as the combined total growth. Moreover, the model provides confidence intervals (CIs) of the different growth estimates that can be used to assess the quality of growth data at the individual level. Basic features of the QEPS-model have been presented at meetings [27–29].

## Methods

### Ethical approval

Ethical approval was obtained from the ethics committee of the University of Gothenburg (91–92/131–93), and individual approval was given by the participants of the 1974 cohort study if they were 18 years or older, or by their legal guardian if they were not old enough to give consent (16 to 18 years of age).

### Subjects – A healthy cohort born in 1974

The data used for analysis was from a community-based, observational growth study the GrowUp1974 Gothenburg study that was conducted in all high schools in Gothenburg, Sweden in 1992 [3]. Longitudinal growth data from healthy individuals born at term (gestational age 37–42 weeks) within this study, together with data from the Swedish Medical Birth Registry, were used to create the Swedish national Growth References used from 2000 [3, 30]. A study group of individuals with longitudinal growth data was selected from the GrowUp1974 population for the present study using the following steps.

1. *Computerized selection* of individuals with height measurements registered for each of the following ages were selected; at birth; as an infant 0 to 9 months (two or more measurements); as a toddler between 9 months and 3.5 years; as a child 3.5 to <6.0 years; as a schoolchild; 6.0 to <9.0 years; as a juvenile 9.0 to <12.0 years; in adolescence 12.0 to <16.0 years; and in adulthood > = 16 years.
2. *Visual growth curve analysis* for confirmation of the growth characteristics of the individuals in the selected study group; see Growth curve analysis section below. This selection reduced the study group with 696 individuals from 2976 to 2280 individuals. The main characteristics of the study group are shown in Table 1.

**Table 1:** Main characteristics of the study group

Variable	Mean	Median	SD	Max	Min
Girls (N = 1139):					
Birth weight, g	3405	3400	468	5670	1620
Birth length, cm	49.9	50.0	2.14	59.0	35.0
Emax^a^, cm	62.86	62.94	2.87	72.29	52.88
Qmax^b^, cm	**97.61**	97.51	7.57	125.11	75.13
Pmax^c^, cm	**12.78**	12.73	3.65	23.60	0.42
Tmax^d^, cm	167.26	167.24	6.04	183.35	145.68
Adult height, cm	167.65	167.6	6.06	183.7	146.5
Boys (N = 1141):					
Birth weight, g	3513	3520	487	5420	1810
Birth lenght, cm	50.5	51.0	2.12	60.0	41.0
Emax, cm	65.08	65.10	2.88	74.82	56.57
Qmax, cm	**104.05**	103.88	8.02	135.32	73.65
Pmax, cm	**17.34**	17.48	3.63	28.85	4.11
Tmax, cm	180.43	180.16	6.62	201.10	157.29
Adult height, cm	180.69	180.4	6.63	201.7	157.3

^a^Gain in adult height in cm due to *E*-function growth

^b^Gain in adult height in cm due to *Q*-function growth

^c^Pubertal gain in adult height in cm due to the *P*-function growth

^d^Modelled total adult height in cm, *T*_*max*_ = *E*_*max*_ + *Q*_*max*_ + *P*_*max*_ − *S*_*max*_ (all estimates by the QEPS-model)

### Mathematical selection criterion (*MathSelect*)

To assess the quality of the fitted individual total height function, *T(age)*, a mathematical selection criterion, *MathSelect,* was used that we developed for the QEPS-model. The *MathSelect* criterion combines information from nine individual variables. Details on how *MathSelect* was constructed can be found in the Additional file 1: Section A2. Two different *MathSelect* values, 0.975 and 0.68 were used for computerized data quality check of the study group. For all figures *MathSelect* 0.975 was used.

### Processing of the data

To construct a longitudinal growth curve for each individual in the present study group, data files were analysed with Matlab software (version 7.13.0 R2012b, The Mathworks). The Matlab Curve Fitting Toolbox was used for regular curve fitting and was customized to perform penalized curve fitting. Individual curves were estimated with 95% CIs for the fitted parameters.

### Growth curve analysis

The quality of the height data were evaluated by visual inspection using QEPS-model-fitted growth charts. The quality of data, and the presence of potential errors that needed further assessment, were evaluated by stepwise observations:

1. *Assessment of outliers*; assessment of individual height data that deviated from the individual growth curve, giving rise to suspicion of input or measurement errors.
2. *Assessment of the adult height;* visual analysis of whether adult height was reached at the last measurement or not.
3. *Comparison* between the new mid-puberty parameter *AgeP50* and visually evaluated age at PHV (AgePHV).

If there was a difference of more than 0.66 years between *AgeP50* and AgePHV, or if observations 1 and 2 above gave rise to uncertainty regarding any data points, the original growth data were reevaluated; if uncertainty remained, the individuals were excluded from the study.

### QEPS variables describing pubertal growth

**Fig. 2:**
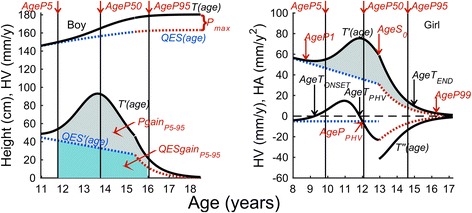
Detailed QEPS pubertal growth estimates. *Left panel*: Showing the height (*upper*) and height velocity (*lower*) graphs of an individual. Subtracting the *Q+E*–*S* (*QES)-*function from *T* gives the pubertal *P-*function. The total gain in adult height due to the specific *P-*function gives the maximum height of the *P*-function, *P*_*max*_ in cm (*P*_*max*_ *= T*_*max*_–*QES*_*max*_). From the *P-*function, onset of pubertal growth can be estimated as the age when 5% (*AgeP5*) of the total *P-*function-estimated gain is reached. *AgeP95* shows the age when 95% of the *P*-function growth is reached. The area under curve, i.e. the *light grey* shaded area between total height velocity (HV) function *T’(age)* and HV function *QES’(age)* = *T’(age)*–*P′(age)*, from *AgeP5* to *AgeP95* is equal to the total area under the curve of pubertal HV *P′(age)* during that time and equal to *Pgain*_*P5–95*._ QEPS-parameter *AgeP50* gives the age where 50% of the total pubertal gain, *P*_*max*_ is reached, so exactly 45% of the light grey shaded area is before and 45% is after *AgeP50*. Total gain in height from *AgeP5* to *AgeP95* is the sum of *Pgain*_*P5–9*5_ and *QESgain*_*P5–95*_ (*light blue shaded area*). *Right panel*: Showing the height velocity (HV, upper) and height acceleration (HA, lower) graphs of an individual. From the *P*-function, onset of pubertal growth can also be estimated as the age when 1% (*AgeP1*) of the total *P*-function-estimated gain is reached. *AgeP*_*PHV*_ is the age at peak height velocity (PHV) from the *P*-function-estimated pubertal growth. *AgeP99* shows the age when 99% of the *P*-function growth is reached. The area under curve, i.e. the light grey shaded area is equal to the total area under the curve of pubertal HV function *P′(age)* and therefore equal to *P*_*max*_. *AgeS0* is the age where the *S-*function starts, which can be seen as a break in the black solid line of the HA function *T”(age)*. From the *total growth curve*, onset of pubertal growth is calculated as the age at minimum HV of the total HV function *T’(age)* at HA function *T”(age)* = 0, *AgeT*_*ONSET*_. Mid-puberty is calculated as the age at PHV from the total growth curve, *AgeT*_*PHV*_ with also *T”(age)* = 0 and end of puberty as the age at which HV had decreased to 1 cm/year, *AgeT*_*END*_

Estimates from the QEPS total curve have the prefix *T (Total),* using the basic additive QEPS- model in which *T(age) = Q(age) + E(age) + P(age) − S(age)* [23]. From the *Total growth curve*, onset of pubertal growth, *AgeT*_*ONSET*_, was calculated as the age at minimum height velocity (HV) of the total height function. Mid-puberty was calculated as the age at PHV from the total growth curve (*AgeT*_*PHV*_) and end of puberty as the age at which HV had decreased to 1 cm/year (*AgeT*_*END*_). The total gain in height (cm) during puberty (*TpubTgain*) was based on growth during the time period *AgeT*_*ONSET*–*END*_ . The total gain in adult height due to the specific *P-*function is estimated by the QEPS-model as the maximum height of the *P-*function, *P*_*max*_ in cm (*P*_*max*_ *= T*_*max*_–*QES*_*max*_). Due to the specific form of the *P-*function, which is a quadratic, logistic function, *P*_*max*_ can be calculated without defining a specific duration of puberty. The *P-*function starts before *AgeT*_*ONSET*_ since the velocity of *T* will not increase until the velocity of the *P-*function increases more than the decreasing velocity of the *QES-*function. If the relative influence of *P-* and *QES-f*unctions during the pubertal time period needs to be calculated, an age point is needed for both *QES-* and *P*-functions. Thus, onset of pubertal growth can be estimated from the *P-*function as the age when 1% (*AgeP1*) or 5% (*AgeP5*) of the total *P-*function-estimated gain was reached. For mid-puberty, we calculated the age when 50% of the *P-*function gain was achieved, *AgeP50,* and the age at PHV from the *P-*function growth curve, *AgeP*_*PHV.*_ In order to identify the end of pubertal growth, we calculated the age when 95% (*AgeP95*) or 99% (*AgeP99*) of the specific pubertal gain was achieved, Fig. 2. The time from *AgeP5* to *AgeP95*, as well as *AgeP1* to *AgeP99* and *AgeT*_*ONSET*_ to *AgeT*_*END*_ gives estimates for the duration of pubertal growth.

**Fig. 3:**
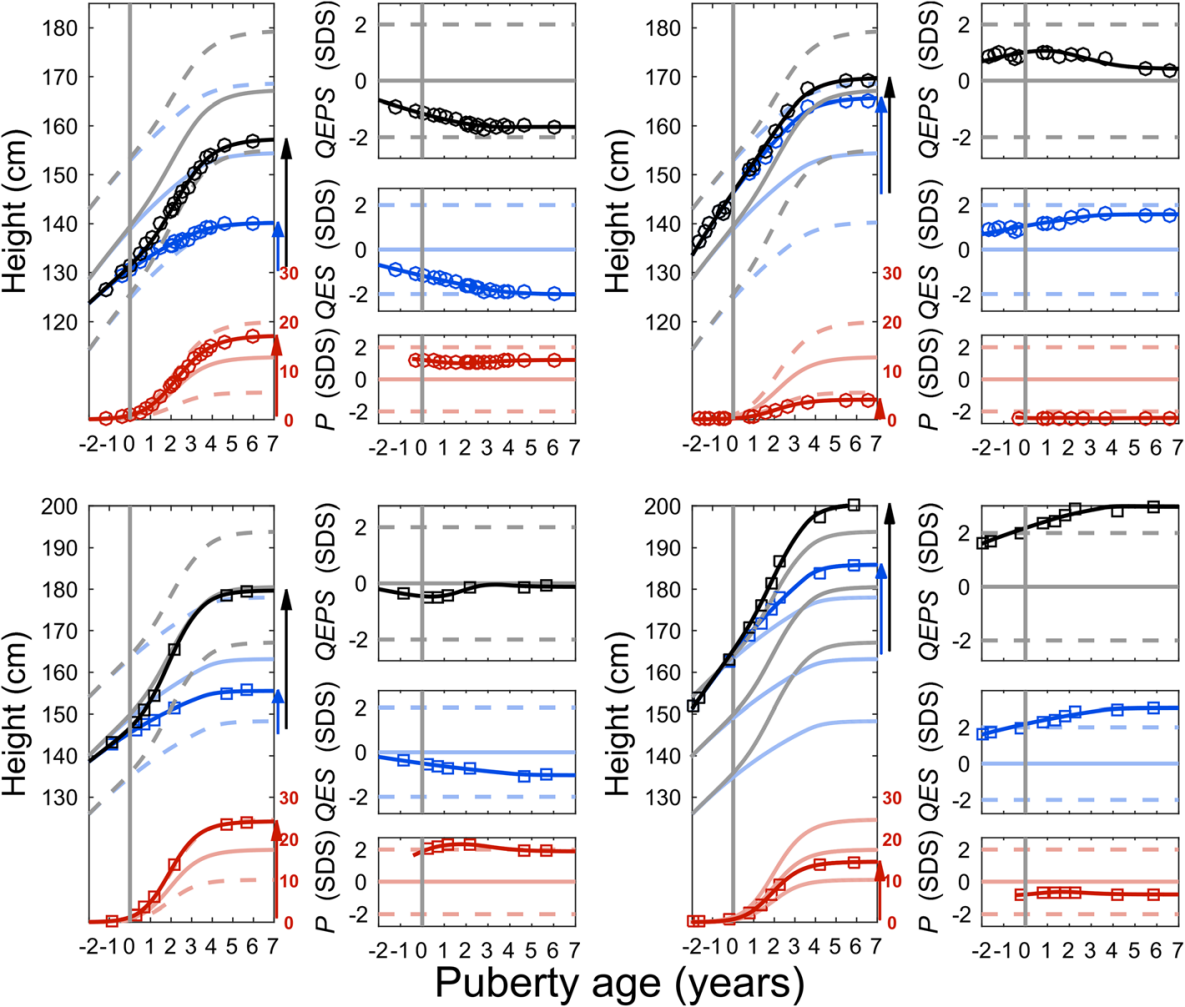
Illustration of four individuals with the pubertal growth divided to the *P-* and *QES-functions*. The increase in height during the pubertal years is shown; to the left, showed in cm, to the right, expressed as SD-scores (SDS). *The vertical solid gray lines* show the individual onset of puberty adjusted to zero by subtracting *AgeP5*. *The upper solid black line* showing the individual height function, *T*, expressed in cm, with the actual measurements indicated as circles (girls) or squares (boys), *the gray solid line* showing the mean *T* values, and *the dotted gray lines *representing ±2 SD-scores. *The solid blue line* showing the individual height function *QES* expressed in cm, *the* *solid light blue line* showing the mean *QES* values, and the *dotted light blue lines* representing ±2 SD-scores. *The solid red line* shows the individual specific *P*-function expressed in cm, *the solid light red line* showing the mean *P* values, and *the dotted light red lines* representing ±2 SD-scores. At the right side are the corresponding *QEPS* height SD-scores for the individual showed in relation to the mean *T*, *QES* and *P-*functions, where the sum of *P* and *QES* give the total growth, *T*. **Upper left panel** shows a girl with a low and decelerating *QES-*function height_SDS_ to −2.0, a *P*-function height_SDS_ around +1.2, resulting in a *T*-function height_SDS_ of −1.7 and an adult height of 157 cm. **Upper right panel** shows a girl with an above average and increasing *QES*-function height_SDS_ from +0.8 to +1.7, a low *P*-function height_SDS_ of −2.2 with a resulting *T*-function height _SDS_ from +1.0 to +0.4 (170 cm). **Lower left panel** shows a boy with a decelerating *QES*-function height_SDS_ from −0.2 two years before puberty to −1.0 at adult height, a *P*-function height_SDS_ around +2.0 with a resulting *T*-function adult height_SDS_ of −0.1 (179 cm). **Lower right panel** shows a boy with a high and accelerating *QES*-function height_SDS_ from +1.8 two years before puberty to +3.0 at the end of puberty, a *P*-function height _SDS_ of −0.7 with a resulting *T*-function height_SDS_ at adult height of +3.0 (200 cm)

In general, pubertal gain can be described as the increase in height of the *T*, *P,* and *QES*-function from *AgeT*_*ONSET*_ until *AgeT*_*END*_, and corresponding decomposition of the total puberty gain in a *P* and a *QES* part: *Tgain* = *Pgain* + *QESgain*. The value of onset and end of pubertal growth can be any selected combination of onset of puberty age, expressed as *AgeT*_*ONSET*_, *AgeP1* or *AgeP5*, with any selected end of puberty age, expressed as *AgeT*_*END*_, *AgeP95* or *AgeP99*. During the selected pubertal period the model can separate the influence of the specific *P-*function from the ongoing *Q-*function, and also their relationship as a ratio with the locations of *AgeT*_*ONSET*_ and *AgeT*_*PHV*_; see Additional file 1: Section A1:3 for more information. Examples of pubertal growth for four individuals are shown adjusted for age at onset of puberty in Fig. 3, and their entire growth according to chronological age in Additional file 1: Figure S1, where the total pubertal growth is expressed in both cm and SD-scores, and is also divided into the *P-* and *QES-*functions. The details of the equations describing pubertal growth can be found in Nierop et al. [23], with complementary information explaining the pubertal period in more detail in the Additional files 1: Section A1.

### Statistical considerations

The measured and calculated variables in the tables are presented as mean, median, standard deviation, maximum and minimum. Lower and upper 95% CIs, skewness and kurtosis computations conducted in order to estimate any departure from the normal distribution are given in the Additional file 1: Tables. These computations were performed using SAS Software 9.3 (SAS Institute Inc., Cary, NC, USA).

## Results

### Pubertal growth estimates

The different pubertal growth estimates are shown in Tables 2 and 3, and in more detail in the Additional file 1: Tables S1 and S2. Differences in the mean between various pubertal estimates are shown below; grouped by the type of measurement; for pubertal duration and pubertal height results are given with the ±1 SD interval of the population in brackets.

**Table 2:** Age in years for pubertal growth estimates

Variable	N	Mean	Median	SD	Max	Min
**Girls:**						
**Onset of puberty**						
**AgeTonset**, age at minimum height velocity of the T-function^a^	1129	9.24	9.19	1.01	12.62	6.37
**AgeP1**, age at 1% of the P-function^b^	1139	8.71	8.68	0.98	12.00	6.09
**AgeP5**, age at 5% of the P-function	1139	**9.86**	9.81	0.97	13.13	7.30
**Mid puberty**						
**AgePHV**, age at visual estimated PHV	1134	11.92	11.88	0.97	15.31	9.35
**AgeTPHV**, age at PHV of the T-function	1129	11.83	11.80	0.96	15.09	9.39
**AgePPHV**, age at PHV of the P-function	1139	12.02	11.98	0.95	15.26	9.51
**AgeP50**, age at 50% of the P-function	1139	12.09	12.06	0.95	15.34	9.59
**End of puberty**						
**AgeP95**, age at 95% of the P-function	1139	14.66	14.65	0.95	17.93	12.23
**AgeP99**, age at 99% of the P-function	1139	16.33	16.34	0.95	19.63	13.91
**AgeTend**, age where the height velocity has decreased to 1 cm/year	1139	15.01	15.03	0.84	18.00	12.85
**Duration**						
Duration between **AgeP5 and AgeP95**	1139	**4.80**	4.78	0.21	5.54	3.44
Duration between **AgeP1 and AgeP99**	1139	7.61	7.58	0.33	8.77	5.45
Duration between **Tonset and Tend**	1129	5.77	5.78	0.50	7.11	3.84
**Boys:**						
**Onset of puberty**						
**AgeTonset**, age at minimum height velocity of the T-function	1141	10.74	10.71	0.98	14.20	7.50
**AgeP1**, age at 1% of the P-function	1141	10.73	10.72	0.97	13.94	7.45
**AgeP5**, age at 5% of the P-function	1141	**11.78**	11.77	0.96	14.98	8.56
**Mid puberty**						
**AgePHV**, age at visual estimated PHV	1136	13.83	13.81	1.00	17.18	10.95
**AgeTPHV**, age at PHV of the T-function	1141	13.66	13.65	0.96	16.84	10.54
**AgePPHV**, age at PHV of the P-function	1141	13.73	13.72	0.96	16.94	10.63
**AgeP50**, age at 50% of the P-function	1141	13.80	13.78	0.96	17.01	10.69
**End of puberty**						
**AgeP95**, age at 95% of the P-function	1141	16.10	16.06	0.97	19.31	13.12
**AgeP99**, age at 99% of the P-function	1141	17.56	17.52	0.98	20.78	14.58
**AgeTend**, age where the height velocity has decreased to 1 cm/year	1141	16.68	16.64	0.90	19.44	14.01
**Duration**						
Duration between **AgeP5 and AgeP95**	1141	**4.32**	4.31	0.22	5.55	3.24
Duration between **AgeP1 and AgeP99**	1141	6.83	6.82	0.34	8.78	5.12
Duration between **Tonset and Tend**	1141	5.94	5.94	0.38	7.62	4.37

^a^Total height function in cm; *T(age) = Q(age) + E(age) + P(age) – S(age),* ^b^Quadratic logistic function describing the pubertal growth spurt *P(age)* in cm

**Table 3:** Estimated heights and pubertal gains in cm

	N	Mean	Median	SD	Max	Min
**Girls:**						
**Onset of puberty**						
**Height at Tonset**, age at minimum height velocity of the T-function^a^	1129	136.15	135.87	7.78	160.45	111.52
**Height at AgeP1**, age at 1% of the P-function^b^	1139	133.34	133.34	7.09	155.41	110.94
**Height at AgeP5**, age at 5% of the P-function	1139	139.47	139.36	6.91	160.72	116.45
**Mid puberty**						
**Height at AgeTPHV**, PHV^c^ of the T-function	1129	152.37	152.32	6.15	169.62	130.45
**Height at P50**, age at 50% of the P-function	1139	154.29	154.07	6.25	171.68	131.92
**End of puberty**						
**Height at AgeP95**, age at 95% of the P-function	1139	165.81	165.77	6.04	181.91	144.26
**Height at AgeP99**, age at 99% of the P-function	1139	166.98	167.02	6.04	183.09	145.41
**Height at Tend**, age where the height velocity has decreased to 1 cm/year	1139	166.24	166.24	6.05	182.39	144.65
**Adult height** (AH)	1139	167.66	167.6	6.06	183.7	146.5
**Height gain**						
Growth in height between **AgeP5 and AgeP95**	1139	**26.34**	26.35	3.81	37.86	12.98
Growth in height between **AgeP1 and AgeP99**	1139	33.64	33.57	4.56	47.33	18.62
Growth in height between **Tonset and Tend**	1129	**30.09**	30.14	5.18	45.56	11.79
Growth in height between **Tonset and AH**	1129	**31.51**	31.54	5.34	46.86	12.73
**Boys:**						
**Onset of puberty**						
**Height at Tonset**, age at minimum height velocity of the T-function	1141	144.60	144.28	7.45	168.17	116.45
**Height at AgeP1**, age at 1% of the P-function	1141	144.53	144.17	7.03	166.56	117.83
**Height at AgeP5**, age at 5% of the P-function	1141	149.76	149.36	6.97	170.80	122.56
**Mid puberty**						
**Height at AgeTPHV**, PHV of the T-function	1141	163.74	163.40	6.54	182.12	138.82
**Height at P50**, age at 50% of the P-function	1141	165.03	164.67	6.58	183.59	140.06
**End of puberty**						
**Height at AgeP95**, age at 95% of the P-function	1141	178.75	178.50	6.59	199.06	155.64
**Height at AgeP99**, age at 99% of the P-function	1141	180.15	179.85	6.62	200.77	157.01
**Height at Tend**, age where the height velocity has decreased to 1 cm/year	1141	179.62	179.35	6.62	200.29	156.49
**Adult height** (AH)	1141	180.69	180.4	6.63	201.7	157.3
**Height gain**						
Growth in height between **AgeP5 and AgeP95**	1141	**29.00**	28.97	3.64	40.17	16.68
Growth in height between **AgeP1 and AgeP99**	1141	35.62	35.55	4.26	49.39	22.17
Growth in height between **Tonset and Tend**	1141	**35.02**	35.08	4.74	48.61	16.41
Growth in height between **Tonset and AH**	1141	**36.09**	36.10	4.89	51.39	17.16

^a^Total height function in cm; *T(age) = Q(age) + E(age) + P(age) – S(age),* ^b^Quadratic logistic function describing the pubertal growth spurt *P(age)* in cm. ^c^Peak height velocity

#### Onset of pubertal growth

Estimates of timing for onset of pubertal growth vary depending on the variable used. For girls, the mean age at onset of puberty as *AgeT*_*ONSET*_ from the total growth curve was 9.24 years, 0.53 years after *AgeP1* and 0.62 years before *AgeP5* from the *P*-function. For boys, there was no difference between *AgeT*_*ONSET*_ (10.74) and *AgeP1* (10.73), whereas *AgeP5* occurred 1.0 years later, Table 2.

The median percentage of the *P-*function reached at the *AgeT*_*ONSET*_ was 2.4% for girls and 1% for boys, Additional file 1: Figure S2.

#### Mid-pubertal growth estimates

The visually estimated age at PHV (AgePHV*)* was compared with the QEPS-calculated *AgeT*_*PHV*_ from the *T*-function and with *AgeP*_*PHV*_ /*AgeP50* from the *P-*function; the mean values of these four estimates of mid-pubertal growth showed minor differences from each other (maximal 3 months), Table 2. The difference in years between AgePHV and *AgeP50* was −0.171 **(**±0.46 SD) for girls and 0.037 **(**±0.36 SD) for boys, Table 2. The median percentage of the *P*-function reached at mid puberty as *AgeT*_*PHV*_ was 43% for girls and 45% for boys*,* Additional file 1: Figure S2, middle panel.

#### End of pubertal growth

**Fig. 4:**
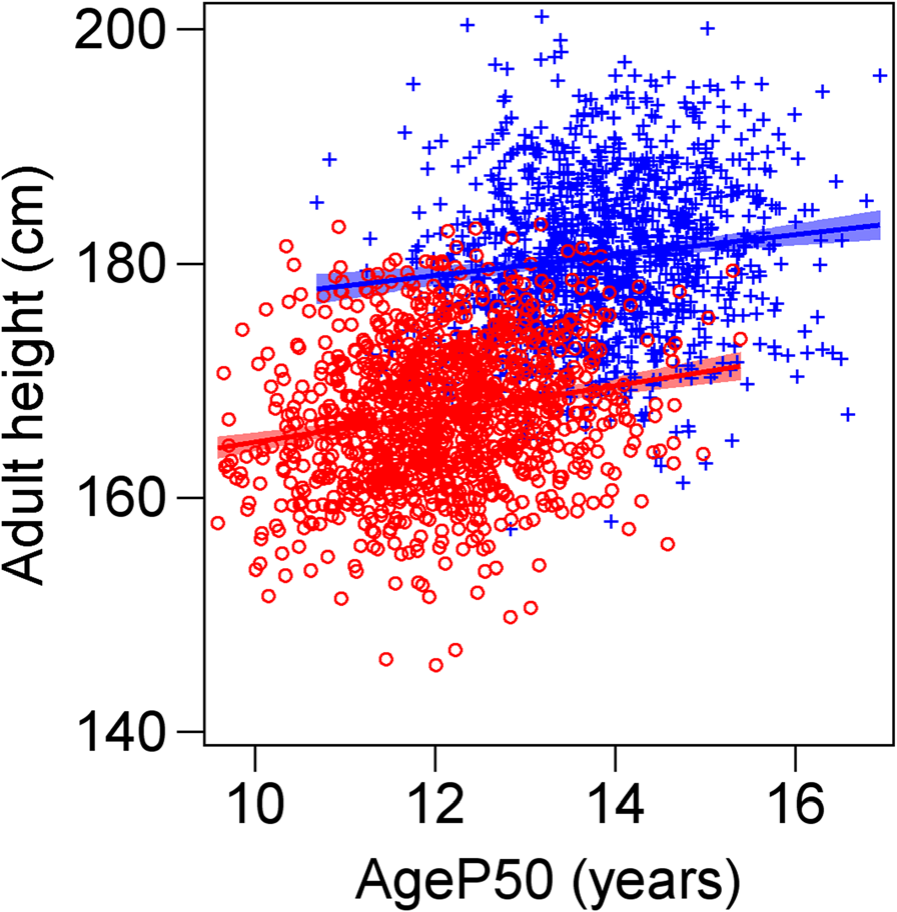
Scatterplot showing the relationship between adult height and *AgeP50.* Age at 50% of the *P*-function (*AgeP50*) for girls (*red circles*) and boys (*blue crosses*) in the study population is related to adult height. For girls; adult height = 152.707+ 1.204 x *AgeP50*, adjusted r^2^ = 0.0363 (i.e. a 1-year delay in onset of puberty will give 1.20 cm taller adult height). For boys; adult height = 168.392 + 0.878 x *AgeP50*, adjusted r^2^ = 0.0154 (i.e. a 1-year delay in onset of puberty will give 0.88 cm taller adult height)

For girls, the mean difference in years between *AgeT*_*END*_ from the total curve and *AgeP95* from the *P-*function was 0.35, and the difference between *AgeT*_*END*_ and *AgeP99* was −1.32. For boys, the corresponding values were 0.58 and −0.88 years, respectively, Table 2. For both genders, taller adult heights were found in individuals with later pubertal growth (later *AgeP50*); however, there was broad individual variation and apparent differences in the distribution of pubertal timing between genders, as seen in Fig. 4. When relating adult height to age at onset of puberty, the pattern was similar for both genders, see Additional file 1: Figure S4; a 1-year delay in the onset of puberty, expressed as *AgeT*_*ONSET,*_ will give an adult height that is taller by 1.2 cm in girls and 0.8 cm in boys. The percentage of *P*-function growth reached at *S*_*0*_ was for girls 74% and for boys 89%, whereas at *AgeT*_*END*_ it was 97% for both genders, Additional file 1: Figure S2 right panel.

#### Duration of pubertal growth

For girls, the mean duration in years for pubertal growth from *AgeP5* to *AgeP95* was 4.80 (4.59–5.01**),** the duration from *AgeP1* to *Age99* was 7.61 (7.28–7.94) and the duration from the total growth curves defined as *AgeT*_*ONSET–END*_ was 5.77 (5.27–6.27).

**Fig. 5:**
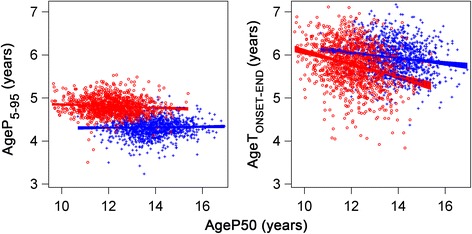
Scatterplot showing the relationship between the duration of pubertal growth and *AgeP50*. *Left panel:* Age from 5% of the pubertal growth (*AgeP5*) to age at 95% of the pubertal growth (*AgeP95*) for girls (*red circles*) and boys (blue crosses) in the study population, represents one estimate for duration of pubertal growth and is related to the timing of *AgeP50*. For girls; *AgeP5* to *AgeP95* = 5.030–0.0184 x *AgeP50*, adjusted r^2^ = 0.0068. For boys; *AgeP5* to *AgeP95* = 4.246 + 0.0052 x*AgeP50*, adjusted r^2^ = −0.0003. *Right panel*: Age from the minimum height velocity before pubertal growth spurt (*AgeT*_*ONSET*_) to the age where the height velocity has decreased to 1 cm/year (*AgeT*_*END*_) girls (*red circles*) and boys (*blue crosses*) represents one estimate for duration of pubertal growth and is related to the timing of *AgeP50*. For girls; *AgeT*_*ONSET*–*END*_ = 7.544–0.147 x *AgeP50,* adjusted r^2^ = 0.081. For boys; *AgeT*_*ONSET*–*END*_ = 6.789–0.0616x*AgeP50*, adjusted r^2^ = 0.0244

The corresponding durations of pubertal growth in years for boys were 4.32 (4.10–4.54), 6.83 (6.49–7.17), and 5.94 (5.56–6.32), respectively, Table 2. A clear gender difference was seen in both timing and duration of pubertal growth when estimates were based on the *P-*function*,* Fig. 5, left panel*,* with not only a later, but also a shorter pubertal growth spurt seen in boys. This is in contrast to the less pronounced gender difference observed when the duration of pubertal growth was based on the total growth curve for the same age-points, Fig. 5, right panel.

### Gain of pubertal growth

From the total growth curve, the mean pubertal gain for girls from *AgeP5* to *Age95* was 26.34 cm (18.74–33.94), and from *AgeP1* to *Age99* it was 33.64 cm (24.52–42.76). For boys, the corresponding pubertal gains were 29.00 (21.72–36.28) and 35.62 cm (27.10–44.14), respectively, Table 3.

**Fig. 6:**
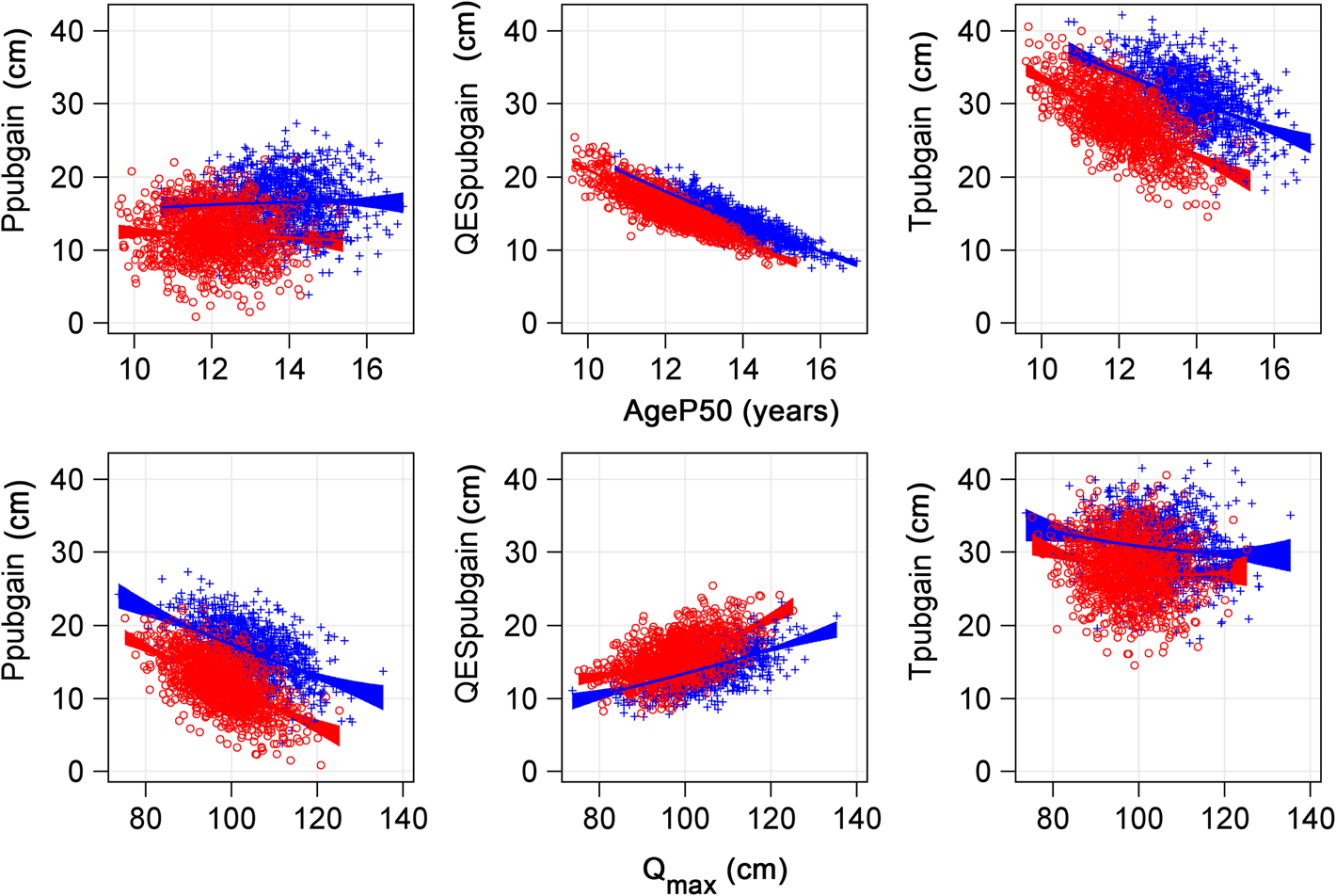
Scatterplot showing relationship between; *Ppubgain* (*left*), *QESpubgain* (*middle*) and *Tpubgain* (*right*), related to *AgeP50* in the upper panels, and related to *Qmax* in the lower panels. Upper left panel: *Ppubgain* as 95% of *P*_*max*_ (the *P*-function of the total growth during puberty) is not related to *AgeP50* (age at 50% of the *P*-function) for girls (*red circles*) and boys (*blue crosses*) in the study population. For girls; *Ppubgain* = 14.393–0.1850× *AgeP50*, adjusted r^2^ = 0.0019. For boys; *Ppubgain* = 15.243 + 0.0917 x *AgeP50*, adjusted r^2^ = −0.0002. Upper middle panel: *QESpubgain* in total height in cm during pubertal years, from 5% of the *P*-function (*AgeP5*) to 100% of *P-*function is related to *AgeP50* (age at 50% of the *P*-function) for girls (*red circles*) and boys (*blue crosses*) in the study population. For girls; *QESpubgain* = 45.430–2.461x*AgeP50;* adjusted r^2^ = 0.769. For boys; *QESpubgain =* 42.899–2.080x*AgeP50* adjusted r^2^ = 0.747. **Upper right panel**: *Tpubgain*, gain in total height in cm during the pubertal years, from 5% of the pubertal growth function (*AgeP5*) to 100% of the pubertal growth function *(AgeP100),* is related to *AgeP50* (age at 50% of the *P*-function) for girls (*red circles*) and boys (*blue crosses*) in the study population. For girls; *Tpubgain* = 59.823–2.647× *AgeP50*, adjusted r^2^ = 0.395. For boys; *Tpubgain* = 58.142–1.988x*AgeP50*, adjusted r^2^ = 0.253. **Lower left panel**: *Ppubgain* as 95% of *P*_*max*_ (the *P*-function of the total growth during puberty) is related to *Q*_*max*_ (gain in adult height in cm due to *Q*-function growth) for girls (*red circles*) and boys (*blue crosses*) in the study population. For girls; *Ppubgain* = 38.704–0.272x*Q*_*max*_, adjusted r^2^ = 0.355. For boys; *Ppubgain* = 40.065–0.226x*Q*_*max*_, adjusted r^2^ = 0.277. **Lower middle panel**: *QESpubgain* in total height in cm during pubertal years, from 5% of the pubertal growth function (*AgeP5*) to 100% of the pubertal growth function *(AgeP100),* is related to *Q*_*max*_ for girls (red circles) and boys (blue crosses) in the study population. For girls; *QESpubgain* = −2.797 + 0.189x*Q*_*max*_ *,* adjusted r^2^ = 0.276. For boys; *QESpubgain =* −2.499 + 0.160x*Q*_*max*_ *,* adjusted r^2^ = 0.303. **Lower right panel**: *Tpubgain*, gain in total height in cm during the pubertal years, from 5% of the pubertal growth function (*AgeP5*) to 100% of the pubertal growth function *(AgeP100)* versus *Q*_*max*_ for girls (red circles) and boys (blue crosses) in the study population. For girls; *Tpubgain* = 35.906–0.0829× *Q*_*max*_, adjusted r^2^ = 0.0228. For boys; *Tpubgain* = 37.566–0.0666× *Q*_*max*_, adjusted r^2^ = 0.0187

The pubertal gain can also be described as what the specific *P-*function adds to the ongoing *QES-*function. The mean pubertal gain, from the *P*-function, *P*_*max*_, was 12.73 cm for girls and 17.34 cm for boys, and was not influenced by the timing of puberty, as seen in Fig. 6, upper left panel (with *Ppubgain*, 95% of *P*_*max*_). However, for both genders, the increase in total height during the pubertal years, *Tpubgain,* appeared to be higher for individuals with earlier puberty compared with those with later puberty, due to differences in the growth from the *QES-*function during these years, Fig. 6, upper middle-right panels. The *Ppubgain* was clearly negatively related to *Q*_*max*_, the higher the *Q*_*max*_, the lesser the *Ppubgain*, also the *Tpubgain* was negatively correlated to *Q*_*max*_, but to a lesser extent as seen in Fig. 6, lower panels.

**Fig. 7:**
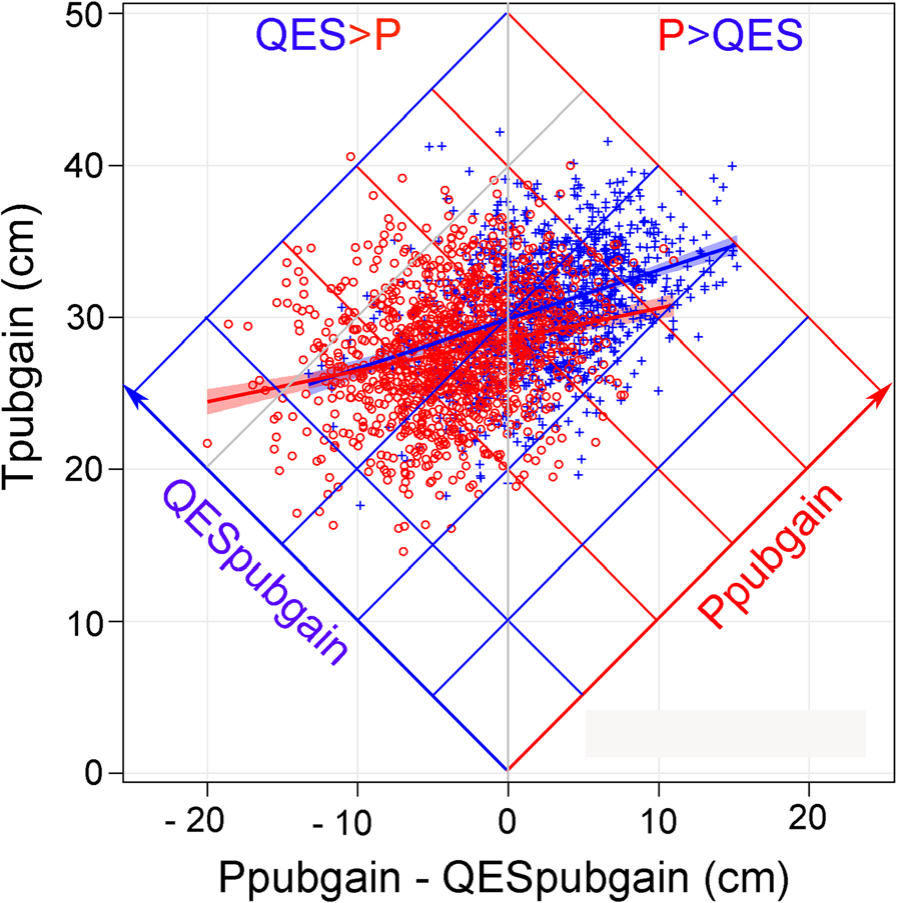
Relationship between *Ppubgain* and *QESpubgain* showing the total growth during puberty. Relationship between *Ppubgain* (=0.95*P_*max*_) and *QESpubgain* expressed as a subtraction on the horizontal axis (cm) and total pubertal gain (cm) on the vertical axis. If *P* gain = *QESpubgain*, then the difference is zero. Different combinations of *Ppubgain* and *QESpubgain* resulting in different total pubertal gain can be evaluated using the transverse lines for each variable. *The oblique* *blue line*, with its transverse blue isolines, represents *QESpubgain*, and *the oblique* *red line*, with its transverse isolines, represents *Ppubgain.* *Red circles* indicate girls and *blue crosses* indicate boys

Using the QEPS-model, pubertal gain can also be shown for each individual both as total gain and divided into the individual components of the *P-*function and the ongoing *QES-*function*,* Fig. 2, left. For the whole study population during the pubertal years, defined as the time period *AgeP5–100* in Fig. 7, growth from the *QES-*function dominated in girls, whereas growth from the *P-*function dominated in boys, but with large inter-individual variations for both genders.

### Tempo-adjusted SD-scores for pubertal age and height

The QEPS-model calculates the age of the individual for all pubertal estimates, which enables these estimates to be compared with the mean of the background population as relative age in deviations from the mean (i.e. standardized age in SD-scores). Thus, instead of showing the age of a child in chronological age, the age at onset of puberty can be visualized according to the mean age (zero) of the internal reference for onset of puberty, i.e. adjusted to pubertal age [23]. With this tempo-correction for the onset of puberty, the QEPS-model enables an individualized reference of pubertal growth in which height_SDS_ can be expressed according to a pubertal tempo-adjusted reference curve as shown in Fig. 3.

Moreover, in the examples of individuals presented in Fig. 3 and in the Additional file 1: Figure S1, (also presenting entire growth vs chronological age), the individual estimates of the different growth functions are presented not only in cm but also in individualized SD-scores.

**Fig. 8:**
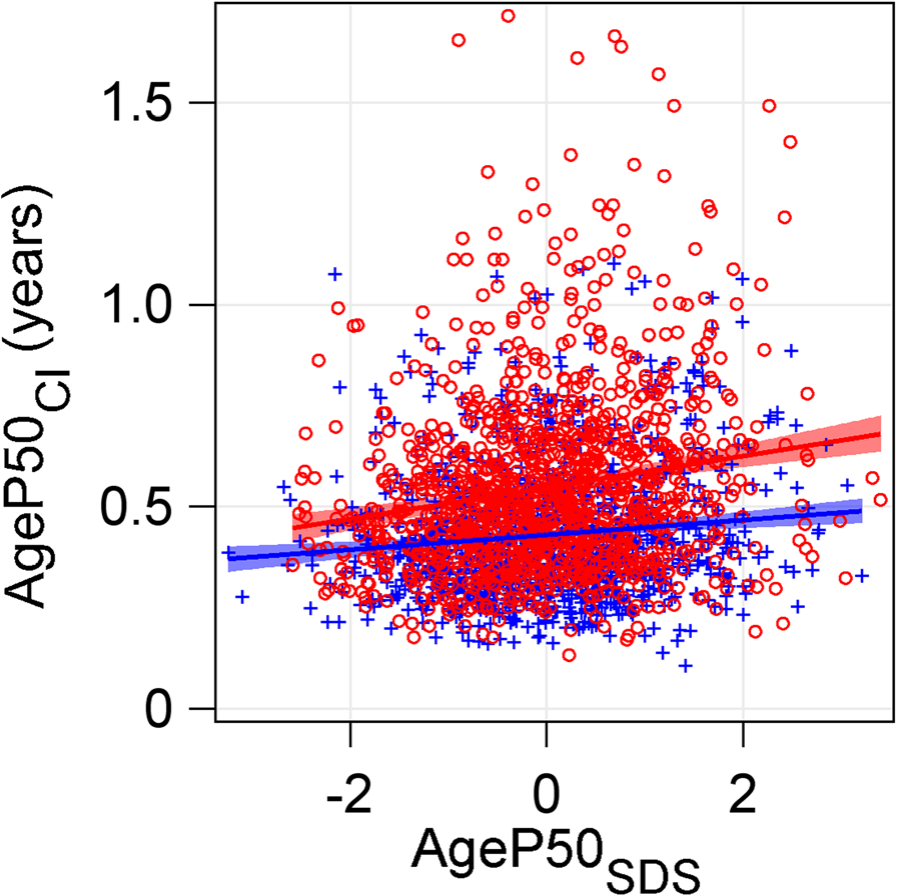
Scatterplot showing the relationship between *AgeP50*_*CI*_ and relative pubertal age (standard deviation scores of *AgeP50*). Relative pubertal age is shown as *AgeP50*_*SDS*_, SD-scores of *AgeP50* (age at 50% of the *P*-function), for girls (*red circles*), for boys (*blue crosses*) in the study population (revealing that early and late puberty can be defined as ±2 SD-scores from mean age of *AgeP50)*, are related to the confidence interval (CI) of *AgeP50 (AgeP50*_*CI*_ *).* For girls; *AgeP50*_*CI*_ = 0.547 + 0.0392 x *AgeP50*_*SDS*_, adjusted r^2^ = 0.0300. For boys; *AgeP50*_*CI*_ = 0.430 + 0.0186 x *AgeP50*_*SDS*_, adjusted r^2^ = 0.0126

In Fig. 8, we show the relationship between the mid-puberty variable, *AgeP50*, and its corresponding CI for standardized mid-puberty (*AgeP50*_*SDS*_). The scatterplot illustrates that the CI increases in those with later pubertal growth, especially in girls. Thus, there is a greater uncertainty in the estimate of mid-puberty for individuals with later pubertal growth.

### Individual CIs for precision and *MathSelect* for quality assurance

From the whole study group, only 49 individuals were removed from the study population/analysis when using *MathSelect* < 0.975, and the absolute differences in pubertal population estimates were small. Kurtosis and skewness decreased only slightly in the *MathSelect* < 0.975 group (excluding CI estimates). In contrast, using *MathSelect* < 0.68, the study group was reduced by 731 individuals, and mostly by affecting skewness estimates, Additional file 1: Tables S3A–C.

**Fig. 9:**
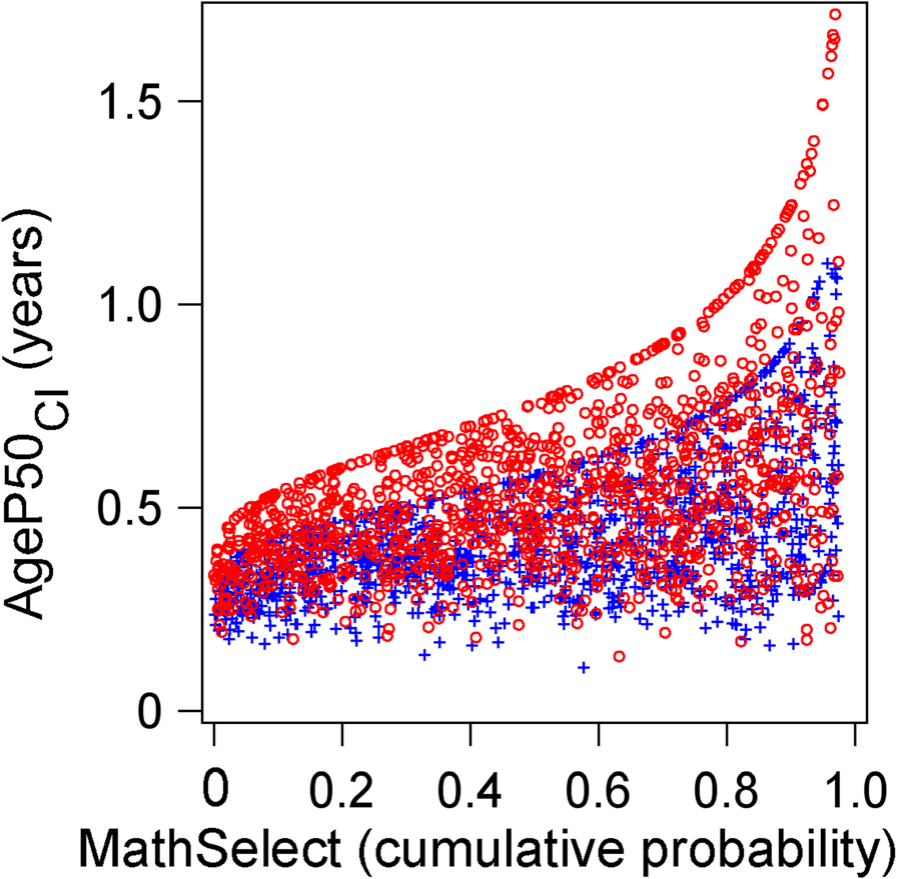
Scatterplot showing the relationship between *AgeP50*_*CI*_ and *MathSelect* values. The confidence interval (CI) for age at 50% of the pubertal growth function (*AgeP50*_*CI*_) is related to the *MathSelect* cumulative probabilities generated for girls (*red circles*) and boys (*blue crosses*) in the study population. The computed *MathSelect* value is giving the expected percentage of a reference group with visually inspected acceptable growth curves having a higher quality than the actual fitted curve.

There was a clear gender difference with higher CIs for girls for all QEPS variables for the onset, middle and end of pubertal growth. Moreover, for both genders, the CIs for variables of onset and end of pubertal growth were broader, than for the estimates of mid-pubertal growth, Additional file 1: Tables S3A–C. These tables also show the resulting lower CIs when reducing the group by using the *MathSelect* function. The relationship between the CIs for *AgeP50* and the *MathSelect* values is shown in Fig. 9. As expected from the modelling procedure, a lower *MathSelect* value corresponds to a lower maximum CI, whereas a higher maximum CI corresponds to a higher *MathSelect* value.

**Fig. 10:**
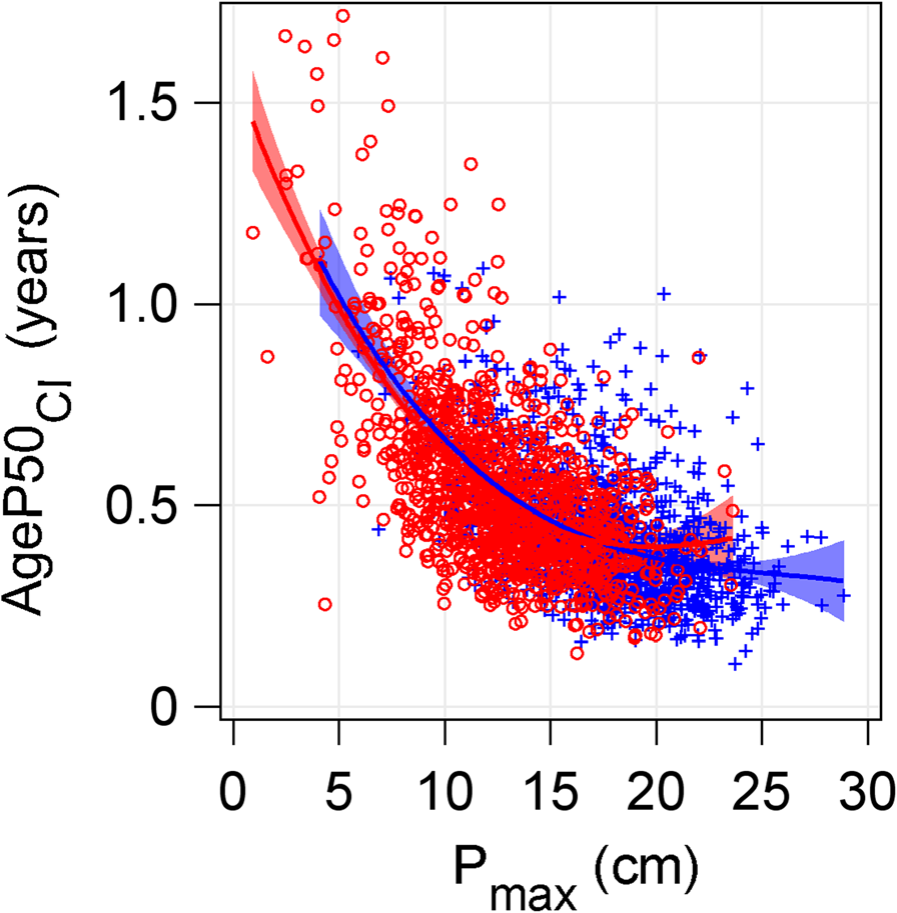
Scatterplot showing the relationship between *AgeP50*_*CI*_ and *P*_*max*_. The relationship between the confidence intervals (CIs) for age at 50% of the pubertal growth function (*AgeP50*_*CI*_) and the total *P-*function height gain (*P*_*max*_) for girls (*red circles*) and boys (*blue crosses*) in the study population is shown. For girls; *AgeP50*_*CI*_ = 1.445–0.1105 x *P*_*max*_ + 0.00290 x *P*_*max*_^2^, adjusted r^2^ = 0.4147. For boys; *AgeP50*_*CI*_ = 1.278–0.0800 x *P*_*max*_ + 0.00171 x *P*_*max*_^2^, adjusted r^2^ = 0.2890

The relationship between the CI for *AgeP50* and the *P-*function height gain (*P*_*max*_) showed a nonlinear correlation; higher CIs were associated with lower *P*_*max*_, Fig. 10. Independent of gender, a pubertal gain below 8 cm, gave a CI of more than 9 months, whereas a gain of at least 14 cm, gave a CI of less than 6 months as shown in Fig. 10. The apparent gender difference was related to the fact that a low *P*_*max*_ was more common in girls.

Additional file 1: Figure S4 shows a QEPS-calculated height velocity graph of an individual with low pubertal height gain, which further illustrates the problems in defining *AgeT*_*PHV*_ and

*AgeT*_*ONSET*_ when the *P*-function is low. To be distinguishable, *P*_*max*_ must be greater than 50% of the CI, which for boys corresponds to a *P*_*max*_ of 2.74 cm and for girls to a *P*_*max*_ of 3.14 cm as seen in Additional file 1: Figure S5.

## Discussion

### Principal findings: QEPS variables for pubertal growth enable new information

The present study, as the first implementation of the QEPS-model to describe pubertal growth, describes the pubertal growth variables generated by the model and their accompanying SD-scores for the population and the individual. Furthermore, the study demonstrates the potential to use these variables to explore human pubertal growth in greater detail than has previously been possible. The variables were calculated for the total growth curve during the pubertal years, and were also separated to provide information on growth specific to puberty, the *P-*function (*Ppubgain*), and growth related to the still ongoing *QES-*function (*QESpubgain*). The *Ppubgain* was found to be independent of age at onset of puberty, whereas the total height gain during puberty, also depending on the *QES-*function, was greater in those with earlier puberty. Moreover, a gender difference was identified, with more *QES-*function growth in girls and more *P-*function growth in boys.

As well as providing robust variables, the QEPS-model is the first growth model to provide individual CIs. Moreover, it allows height SD-score estimations during puberty to be expressed in relation to an individualized tempo-adjusted reference. This is a major achievement as it allows relative growth during the pubertal years to be expressed at any time-point; previous models have only been able to present total pubertal gain from pre-puberty to adult height which has limited in depth analysis regarding pubertal growth [8]. By applying the QEPS-model to longitudinal growth data, we have identified new mathematical variables that are linked to specific time-points and which can be used to describe pubertal growth in detail, thus enabling comparison of growth patterns between individuals and populations. A practical advantage of using the QEPS-model compared to other growth models is that it automatically describes a wide variety of growth-related variables without relying on visual inspection of growth data; thus, the model is not subject to the estimation errors that can occur when relying on visual assessments.

### Onset of puberty

The QEPS-model gives different time-points that differ from each other for onset of puberty; from the total growth curve as well as from the specific pubertal growth curve. Based on the specific *P-*function, *(AgeP1),* the onset of puberty was estimated to be 1.4 years earlier than in previous studies of pubertal growth in Scandinavian populations. Similarly, the onset of puberty was 0.9 years earlier when estimated based on the total growth curve*, AgeT*_*ONSET*_ [2, 31]. Our findings are consistent with other studies using mathematical models [17, 19, 32], which typically result in earlier estimates of pubertal onset compared with studies using visual estimates of the onset of puberty [13]. Future studies may show how the *AgeP1* and *AgeT*_*ONSET*_ estimates correlate in the individual with the time when gonadal steroids start to increase during the nighttime [33, 34] which is another way of identifying onset of puberty. In fact, *AgeP5* (9.9 years) at onset of puberty in girls is approximately equal to the onset of puberty in other Scandinavian studies; our results were only 0.24 years earlier than in the Finnish study [31] and 0.34 years earlier than in the Danish study [2], both of which used a visually defined onset of puberty. For boys, a consistency between *AgeP5* (11.8) and onset of puberty in other Scandinavian studies was even greater than for girls, with differences varying from −0.18 to +0.24 years [2, 31, 35].

### Mid-pubertal growth estimates

Mid-puberty, expressed as visual PHV, has so far been the main estimate of pubertal timing used in the literature [2, 6, 7, 17–19, 21, 31]. Here we compared three new estimates of mid-pubertal growth generated by the QEPS-model; from the total growth curve *AgeT*_*PHV*_ *,* and from the *P-*function growth curve *AgeP*_*PHV*_ and *AgeP50,* and found their mean values to be close to each other; both of the QEPS mathematically calculated variables of age at PHV were similar to visual age at PHV. For both boys and girls, we found the strongest correlation to be between *AgeP50* and visual age at PHV [3]; at a population level, the mean difference between *AgeP50* and visual PHV for boys was only 13 days and for girls 62 days. This suggests *AgeP50* to be a variable that could be considered for use to identify age at mid-puberty in future studies of pubertal growth.

### End of puberty and duration of pubertal growth

The QEPS-model enables us to estimate the end of pubertal growth. In fact, there is no other growth model today that can precisely estimate the end of growth [12, 17–19, 21, 22]; therefore, little attention has been paid to the end of pubertal growth. In this work, we introduced 95 and 99% of the *P*-function curve (*AgeP95* and *AgeP99*, respectively), as well as the end of the total curve, *AgeT*_*END*,_ as possible variables for defining the end of pubertal growth. Due to the lack of variables with which to estimate the end of pubertal growth, the duration of puberty has rarely been included in studies of pubertal growth; the study by Taranger & Hägg [15] is one of few exceptions; however, they employed only visual inspection to identify an point corresponding to *AgeT*_*END*_. Using the new variables presented here, the duration of pubertal growth can be expressed for individuals and study populations in future research.

### Total pubertal gain

The shape-invariant QEPS-model is the first growth model that can calculate and describe the specific pubertal height gain together with the total height gain during puberty at an individual level. The specific pubertal height gain was found to be independent of the age at onset of puberty. This was in contrast to the total height gain during the pubertal years which was greatest in those with an early onset of puberty, as reflected in the model by more growth associated with the *QES-*function than the *P-*function. It has been debated whether or not adult height is dependent on the timing of puberty [13, 36, 37]. The results of the present study confirm that there is an impact of a delay in onset of puberty, with a taller adult height in both boys and girls who experienced a later onset of puberty and a later *AgeP50*; in fact a 1-year delay gave approximately a 1 cm greater adult height. For some individuals, mainly girls, the estimated pubertal gain was so low that it was not possible to calculate either *AgeT*_*PHV*_ or *AgeT*_*ONSET*_ from the total growth curve. We can now also define the specific component of the pubertal growth spurt, and using CIs we are also able to assess the accuracy of the estimated measurements. This represents an advance on what was possible using the previous ICP- and SITAR-models [12, 21, 22]. The relation between *Ppubgain* and *QESpubgain* varies between genders, but also between individuals, with more *QESpubgain* in those with earlier puberty.

### Tempo-adjusted individualized reference gives SD-scores for pubertal growth

The relative age at onset of puberty is of major interest to both researchers and clinicians because of the great variation between individuals in biological maturity during the pubertal years [6]. So far, only changes in total pubertal height gain have been described with SD-scores. For the analysis of individual growth patterns during puberty, Tanner et al. constructed “tempo-conditional” height-velocity curves [6], for individuals with early, average or late puberty, which were applied and further modified in recently updated growth charts for the UK [38], whereas Karlberg superimposed pubertal growth curves adjusted for the timing of the mean age of PHV [12]. In the present study, we describe the individual pubertal growth curve in relation to a pubertal reference adjusted to both time and to age. We calculate and present numerically the relative pubertal age for each individual in comparison to the mean for the population /reference curve, presented as SD-scores. Up to now, only the shape-invariant SITAR-model can adjust for individual tempo, amplitude, and size of total pubertal growth [22]. It is important to note that in contrast to the SITAR-model which only describes growth during the pubertal years, the QEPS-model can describe growth from birth until adult height, where growth during the pubertal years is based on two different additive functions that separate growth from the continuously ongoing *QES-*function from growth by the specific *P-*function. These different growth-functions are probably regulated by different factors/hormones, and will therefore be of considerable use when searching for/identifying regulatory factors for growth. Thus, these QEPS-variables will enable us to make a more precise description of individual growth during puberty, related to the individual timing of puberty, as well as to the balance between the different growth functions of the model. However, not only pubertal, but also good prepubertal data is required for calculating the *QES-*function as well as the *P*-function with good accuracy. Height expressed in SD-scores versus a tempo-adjusted height reference will serve as an individualized reference that is unique for this model.

### Quality markers of individual and population growth data

Using CIs as a quality marker for growth in an individual has to our knowledge not been done before, despite the almost universal use of CIs to show the quality of data. Information on CIs makes it possible to visualize the quality of data for each individual; thereby providing information on the number of measurements that are required during the different periods of growth for the construction of a reliable growth curve *at the individual level*.

On the population level, data quality estimation by *MathSelect* enables the quality of growth data to be graded, and selection with the *MathSelect* function is easy and reproducible. We found it to be a useful instrument for identifying individuals with missing or unreliable height values; findings that were confirmed by visual inspection of the computerized growth charts of these individuals. Thus, using the *MathSelect* function can be a method for checking the quality of pubertal growth data in future studies, especially when it comes to the assessment of outliers and individuals for whom there may be measurement/input errors.

### Limitations of the study

The current study presents results on pubertal growth that are specific for the population studied. Thus, the exact numerical values of the different pubertal variables cannot be generalized to pubertal growth in children born in other countries or during other times, with different tempo of secular changes. Instead, it can be used as a baseline for comparisons with studies in the future using either old or new data.

The implementation of the QEPS-model in this study was based on the same study group as the development of the model [3, 30], which may also be regarded as a limitation. However, the model was developed based on mean values, whereas in this present study, the implementation and analyses were done at an individual level, for the 2280 individuals included.

As a model for puberty, it is also important to note that the QEPS-model relies only on information about growth, without any information on the hormonal changes and/or other manifestations that characterise this period of development. Future studies in individuals should be undertaken in order to correlate the pubertal growth variables from the QEPS-model with both hormonal changes [33, 34, 39, 40] and secondary sexual characteristics [41–43] in order to link the four growth functions with underlying biological processes.

## Conclusion

During puberty, the QEPS-model can mathematically delineate the total growth curve as well as identify growth resulting from both the specific pubertal growth *P-*function and the continuation of the prepubertal growth *QES-*function, using four shape-invariant growth functions, with four height-scale and two time-scale parameters. Different variables estimating the onset, middle and end of pubertal growth will enable us to collect measures of both the duration of, and height gain associated with, the *P*-function in relation to total growth during puberty. The QEPS-model is the first growth-model that expresses the timing and amount of pubertal growth in individual SD-scores, thereby indicating both the tempo and the amount of growth at any time-point for the individual in relation to a reference population. Moreover, all pubertal variables are described with individual CIs for the first time, allowing both the population and individual measurements to be more precisely evaluated.

New insights have been achieved for gender-specific pubertal growth; the specific pubertal height gain was found to be independent of age at onset of puberty, whereas the total height gain during puberty, also depending on the *QES-*function, was greater in those with earlier puberty. Moreover, a gender difference was identified, with more *QES-*function growth in girls than boys and more *P-*function growth in boys than girls. The pubertal growth variables from the QEPS-model implemented in this study, will enable us to standardize methods to assess, describe and compare pubertal growth in different populations and patient sub-groups, and will also serve as a tool for gaining new insights into pubertal growth.

## Additional file

Additional file 1: The first two sections explain pubertal variables of the QEPS-model in more detail in texts, figures and tables for the general pubertal growth, section *A.1:1* and the individual variation in pubertal growth, section *A.1:2,* and the *PQ*_*-ratio*_ in *A1:3*. The construction of the mathematical selection criterion, *MathSelect*, is described in section *A.2:1*, in texts, figures and tables, and extreme possible values of the nine input variables corresponding with *MathSelect* values are computed in section *A.2:2*. (ZIP 4423 kb)

## Abbreviations

*AgeP1*: age at which 1% of the *P*-function growth is reached
*AgeP5*: age at which 5% of the *P*-function growth is reached
*AgeP50*: age at which 50% of the *P*-function growth is reached
*AgeP95*: age at which 95% of the *P*-function growth is reached
*AgeP99*: age at which 99% of the *P*-function growth is reached
AgePHV: visually estimated age at peak height velocity
*AgeT*_*END*_: age at the end of puberty where the HV has decreased to 1 cm/y for function *T’(age)*
*AgeT*_*ONSET*_: age at minimum height velocity of the *T-*function at start of the pubertal growth
*AgeT*_*PHV*_: age at Peak Height Velocity of the *T-*function
*CDF*: cumulative distribution function
CI: confidence interval
*E*: negative exponential growth function of age *E(age)* in cm
*E*_*heightscale*_: individual height scale ratio, modifying the height scale of the *E*-function growth, with *E*_*heightscale*_ = *E*_*max*_ / *mE*_*max*_
*E*_*max*_: gain in adult height in cm due to *E*-function growth
*E*_*timescale*_: individual time scale ratio; modifying the time scale of the *E*-function growth*,* and therefore inversely related to the tempo of *E*. The origin is at *t*_0_, the age when length is theoretically zero, *E(t*_0_ *)* = 0, *Q(t*_0_ *)* = 0
HA: height acceleration
Height_SDS_: Height position related to the reference standard deviation score
HV: height velocity
*MaxCDF*: individual maximum cumulative probability out of nine *MathSelect* step one cumulative distribution functions: max(*F*_*TSDerror*_(*T*_*SDerror*_), *F*_*AgeP50CI*_(*AgeP50*_*CI*_), *F*_*PheightscaleCI*_(*P*_*heightscalePCI*_), *F*_*PtimescaleCI*_(*P*_*timescaleCI*_), *F*_*SPheightinterceptCI*_(*SP*_*heightinterceptCI*_), *F*_*SPheightscaleCI*_(*SP*_*heightscaleCI*_), *F*_*EtimescaleCI*_(*E*_*timescaleCI*_), 2*abs(*F*_*ΔTmaxAH*_(*ΔTmaxAH*) − 0.5), *F*_*Penalty*_(*Penalty*))
*MathSelect*: criterion for assessing the quality of the fitted total individual height function *T(age)* by combining nine parameters: *T*_*SDerror*_, *AgeP50*_*CI*_ *, P*_*heightscalePCI*_ *, P*_*timescaleCI*_ *, SP*_*heightinterceptCI*_ *, SP*_*heightscaleCI*_ *, E*_*timescaleCI*_ *, ΔTmaxAH* and *Penalty*.
*P*: quadratic logistic function describing the pubertal growth spurt *P(age)* in cm
*P*_*AUC*_: pubertal area under the curve of pubertal height velocity *P′(age)*, equals maximum *P*_*max*_ of the pubertal height function *P(age)*
*Penalty*: penalty ratio bias / (error + bias) from fitting *T(age)*
*Pgain*_*Px%-y%*_: gain in total height in cm due to the pubertal growth of the *P*-function from x% till y% of the *P*-function, so *Pgain*_*P5–95*_ is the *Pgain* from *AgeP5* to *AgeP95.*
*P*_*heightscale*_: individual height scale ratio, modifying the height scale of the *P*-function, with *P*_*heightscale*_ = *P*_*max*_ / *mP*_*max*_
PHV: Peak height velocity
*P*_*max*_: pubertal gain in adult height in cm due to the *P*-function growth*,* equal to *P*_*AUC*_
*Ppubgain*: *Pgain*_*P5–100*_ = *P*_*max*_ – *P(AgeP5) = 0.95*P*_*max*_
*P*_*timescale*_: individual time scale ratio, modifying the time scale of the *P*-function and is therefore inversely related to the tempo of *P*. The origin is at *AgeP50*, the age at which 50% of the individual *P*-function is reached
*Q*: quadratic growth function of age *Q(age)* in cm
*QES(AgeP5) T(AgeP5)*: *0.05 * P*_*max*_ *.*
*QESgain*_*Px%-y%*_: gain in total height in cm due to the pubertal growth of the *QES*-function from x% till y% of the *P*-function, so *QESgain*_*P5–95*_ is the *QESgain* from *AgeP5* to *AgeP95*
*QES*_*max*_: *T*_*max*_ – *P*_*max*_
*QESpubgain*: *QESgain*_*P5–100*_ = *QES*_*max*_ – *QES(AgeP5)*
*Q*_*max*_: gain in adult height in cm due to *Q*-function growth
*S*: stop function *S(age)* in cm, stopping the *Q*-function growth at the end of growth
SD: standard deviation
*T*: total height function in cm; *T(age)* = *Q(age)* + *E(age)* + *P(age)* – *S(age)*
*Tgain*_*Px%-y%*_: gain in total height in cm due to the pubertal growth of the *T*-function from x% till y% of the *P*-function, so *Tgain*_*P5–95*_ is the *Tgain* from *AgeP5* to *AgeP95.*
*T*_*max*_: modelled total adult height in cm, *T*_*max*_ = *E*_*max*_ + *Q*_*max*_ + *P*_*max*_ − *S*_*max*_
*Tpubgain*: *Tgain*_*P5–100*_ = *T*_*max*_ – *T(AgeP5)*
*TpubTgain*: *T(AgeT*_*END*_ *)* – *T(AgeT*_*ONSET*_ *)*

## References

[1] Bogin B (1999). Evolutionary perspective on human growth. Annu Rev Anthropol.

[2] Aksglaede L (2008). Forty years trends in timing of pubertal growth spurt in 157,000 Danish school children. PLoS One.

[3] Wikland KA (2002). Swedish population-based longitudinal reference values from birth to 18 years of age for height, weight and head circumference. Acta Paediatr.

[4] Delemarre-van de Waal HA (2005). Secular trend of timing of puberty. Endocr Dev.

[5] Parent AS (2003). The timing of normal puberty and the age limits of sexual precocity: variations around the world, secular trends, and changes after migration. Endocr Rev.

[6] Tanner JM, Whitehouse RH (1976). Clinical longitudinal standards for height, weight, height velocity, weight velocity, and stages of puberty. Arch Dis Child.

[7] Tanner JM, Whitehouse RH, Marubini E, Resele LF (1976). The adolescent growth spurt of boys and girls of the Harpenden growth study. Ann Hum Biol.

[8] Karlberg J (2003). Pubertal growth assessment. Horm Res.

[9] Karlberg P (1976). I. Physical growth from birth to 16 years and longitudinal outcome of the study during the same age period. Acta Paediatr Scand Suppl.

[10] Persson I (1999). Influence of perinatal factors on the onset of puberty in boys and girls: implications for interpretation of link with risk of long term diseases. Am J Epidemiol.

[11] Liu YX, Wikland KA, Karlberg J (2000). New reference for the age at childhood onset of growth and secular trend in the timing of puberty in Swedish. Acta Paediatr.

[12] Karlberg J (1987). Analysis of linear growth using a mathematical model. II. From 3 to 21 years of age. Acta Paediatr Scand Suppl.

[13] Hagg U, Taranger J (1991). Height and height velocity in early, average and late maturers followed to the age of 25: a prospective longitudinal study of Swedish urban children from birth to adulthood. Ann Hum Biol.

[14] Albertsson-Wikland K (2014). Growth hormone dose-dependent pubertal growth: a randomized trial in short children with low growth hormone secretion. Horm Res Paediatr.

[15] Taranger J, Hagg U (1980). The timing and duration of adolescent growth. Acta Odontol Scand.

[16] Ledford AW, Cole TJ (1998). Mathematical models of growth in stature throughout childhood. Ann Hum Biol.

[17] Preece MA, Baines MJ (1978). A new family of mathematical models describing the human growth curve. Ann Hum Biol.

[18] Sayers A, Baines M, Tilling K (2013). A new family of mathematical models describing the human growth curve-erratum: direct calculation of peak height velocity, age at take-off and associated quantities. Ann Hum Biol.

[19] Largo RH (1978). Analysis of the adolescent growth spurt using smoothing spline functions. Ann Hum Biol.

[20] Karlberg J (1987). Analysis of linear growth using a mathematical model. I. From birth to three years. Acta Paediatr Scand.

[21] Karlberg J (1989). A biologically-oriented mathematical model (ICP) for human growth. Acta Paediatr Scand Suppl.

[22] Cole TJ, Donaldson MD, Ben-Shlomo Y (2010). SITAR--a useful instrument for growth curve analysis. Int J Epidemiol.

[23] Nierop AF (2016). Modelling individual longitudinal human growth from fetal to adult life QEPS I. J Theor Biol.

[24] Wikland KA (2000). Validated multivariate models predicting the growth response to GH treatment in individual short children with a broad range in GH secretion capacities. Pediatr Res.

[25] Dahlgren J (2007). Models predicting the growth response to growth hormone treatment in short children independent of GH status, birth size and gestational age. BMC Med Inform Decis Mak.

[26] Kristrom B (2009). The first-year growth response to growth hormone treatment predicts the long-term prepubertal growth response in children. BMC Med Inform Decis Mak.

[27] Holmgren A (2013). New puberty growth model for estimation of individual pubertal growth parameters and their precision. Horm Res in Ped.

[28] Holmgren A (2013). New puberty growth model for estimation of age for peak height velocity compared with a manual method. Hormone research in paediatrics.

[29] Hermanussen M, et al. *Adolescent growth: genes, hormones and the peer group. Proceedings of the 20th Aschauer Soiree, held at Glücksburg castle, Germany, 15th to 17th* November 2013. Pediatric Endocrinol Rev. 2014;11(3):341–53. 24716402

[30] Niklasson A, Albertsson-Wikland K (2008). Continuous growth reference from 24th week of gestation to 24 months by gender. BMC Pediatr.

[31] Wehkalampi K (2011). Advanced pubertal growth spurt in subjects born preterm: the Helsinki study of very low birth weight adults. J Clin Endocrinol Metab.

[32] Martin DD, Hauspie RC, Ranke MB (2005). Total pubertal growth and markers of puberty onset in adolescents with GHD: comparison between mathematical growth analysis and pubertal staging methods. Horm Res.

[33] Albertsson-Wikland K (1997). Twenty-four-hour profiles of luteinizing hormone, follicle-stimulating hormone, testosterone, and estradiol levels: a semilongitudinal study throughout puberty in healthy boys. J Clin Endocrinol Metab.

[34] Ankarberg-Lindgren C, Norjavaara E (2008). Twenty-four hours secretion pattern of serum estradiol in healthy prepubertal and pubertal boys as determined by a validated ultra-sensitive extraction RIA. BMC Endocr Disord.

[35] Silventoinen K (2008). Genetics of pubertal timing and its associations with relative weight in childhood and adult height: the Swedish young male twins study. Pediatrics.

[36] Lindgren G (1978). Growth of schoolchildren with early, average and late ages of peak height velocity. Ann Hum Biol.

[37] Tanner JM, Davies PS (1985). Clinical longitudinal standards for height and height velocity for north American children. J Pediatr.

[38] http://www.rcpch.ac.uk/growthcharts. Accessed 5 Mar 2017.

[39] Albin AK (2012). Estradiol and pubertal growth in girls. Horm Res Paediatr.

[40] Albin AK, Norjavaara E (2013). Pubertal growth and serum testosterone and estradiol levels in boys. Horm Res Paediatr.

[41] Marshall WA, Tanner JM (1969). Variations in pattern of pubertal changes in girls. Arch Dis Child.

[42] Marshall WA, Tanner JM (1970). Variations in the pattern of pubertal changes in boys. Arch Dis Child.

[43] Prader A (1966). Testicular size: assessment and clinical importance. Triangle.

