# Supplementary material for: Insight into human pubertal growth by applying the QEPS growth model
Source: BMC Pediatr. 2017 Apr 19;17:107. doi: 10.1186/s12887-017-0857-1 (PMC5395895; doi:10.1186/s12887-017-0857-1)
Supplement: Additional file 1: — The first two sections explain pubertal variables of the QEPS-model in more detail in texts, figures and tables for the general pubertal growth, section A.1:1 and the individual variation in pubertal growth, section A.1:2, and the PQ -ratio in A1:3. The construction of the mathematical selection criterion, MathSelect, is described in section A.2:1, in texts, figures and tables, and extreme possible values of the nine input variables corresponding with MathSelect values are computed in section A.2:2. (ZIP 4423 kb) [file 12887_2017_857_MOESM1_ESM.zip › QEPSPUB 20170414 Additional file.docx]

**Insight into human pubertal growth by applying the QEPS growth model**

**Additional file**

The first two sections explain pubertal variables of the QEPS-model in more detail for the typical mean pubertal growth, section *A.1:1* and the individual variation in pubertal growth, section *A.1:2,* and the *PQ_ratio_* in *A1:3*. The construction of the mathematical selection criterion, *MathSelect*, is described in section *A.2:1*, and extreme possible values of the nine input variables corresponding with *MathSelect* values are computed in section *A.2:2*.

**Methods**

**A1 Pubertal growth spurt**

**A.1:1 Typical mean pubertal growth spurt**

In order to explain pubertal QEPS variables in more detail, an investigation of the QEPS-model including height velocity (HV) and height acceleration (HA) is needed. In Figure 2 we display the typical mean model values for total height *T(age)* with and without pubertal function *P(age)*. The corresponding total HV-function *T'(age)* and HA-function *T''(age)* with and without pubertal HV- and HA-function are also shown. The gain in adult height due to the pubertal growth is estimated in the QEPS-model with the asymptotic maximum height of *P(age)*, *P_max_*_,_ and is equal to the total area of pubertal HV-function *P'(age)*, *P_TAUC_*, indicated with the shaded area in Figure 2, right panel. The variables *AgeP1*, *AgeP5*, *AgeP50*, *AgeP95,* and *AgeP99* give the ages at which 1%, 5%, 50%, 95%, and 99%, respectively; of the total P-function growth, specific pubertal gain, in adult height *P_max_* is reached. The age at PHV of the total height function (*AgeT_PHV_*) is at the local maximum of HV-function *T'(age)* and is found at the age where HA-function *T''(age)* is decreasing and equal to zero, so *T''(AgeT_PHV_)*=0. Likewise, the age at minimum HV of the total height function (*AgeT_ONSET_* =*AgeT_MHV_*) is at the local minimum of the HV-function *T'(age)*, and is found at the age where HA *T''(age)* is increasing and equal to zero, so *T''(AgeT_ONSET_)*=0. At *AgeS_0_*, the age at the start of stop function *S(age)*, when 74% of the *P-*function is reached for each girl and 89% for each boy, HA-function *T''(age)* makes a discontinuous jump in decelerating total growth speed. The dotted (blue) line marking the HA-function *T''(age)* without pubertal HA-function *P''(age)* is constant before *AgeS_0_*, because *T''(age)* –*P''(age)*= *Q''(age)+E''(age)*=2*Q_quadr_*. The last equation is almost exact because in the puberty age range *E''(age)*=0 and *Q''(age)* is always equal to two times the quadratic polynomial coefficient *Q_quadr_* of *Q(age)*. The constant 2*Q_quadr_*, modelling a constant HV decrease per year, is equal to −5.03mm/y^2^ for girls and −4.25 mm/y^2^ for boys in the typical mean model. The PHV of the puberty HV-function *P'(age)*, *AgeP_PHV_*, is located at the age where HA-function *T''(age)* is decreasing and equal to 2*Q_quadr_*, so *T''(AgeP_PHV_)*=2*Q_quadr_* and *P''(AgeP_PHV_)*=0. The HV-function *P'(age)* is only slightly asymmetrical, since *AgeP_PHV_* is for all boys and girls at the age when 48% of the *P*-function growth is reached, just before *AgeP50*. The variable *AgeT_END_* marks the age where the total HV-function *T'(age)* has decreased to 1 cm/year (10 mm/year on the y-axis).

**A.1:2 Individual variations in pubertal growth**

There are four fitted QEPS variables influencing the individual variation in pubertal growth: heigh-scale ratio *P_heightscale_* (modifying the height scale of the *P*-function, with *P_heightscale_* = individual maximum *P_max_* / typical mean maximum *mP_max_*), mid-puberty *AgeP50* (the age at which 50% of the *P*-function growth is reached), time-scale ratio *P_timescale_* (modifying the time-scale of the *P*-function and therefore inversely related to the tempo of *P*) and prepubertal height-scale ratio *Q_heightscale_* (modifying the height-scale of the *Q*-function growth with *Q_heightscale_* = *Q_max_* / *mQ_max_*). All individual *AgeP* and *AgeS* variables in the QEPS-model are exactly related to their corresponding typical mean *mAgeP* and *mAgeS* variables by the time-scale variable *P_timescale_* =(*AgeP* –*AgeP50*)/(*mAgeP* –*mAgeP50*), which is constant within each individual. The locations of the individual *AgeT* variables have a more complex relationship with the pubertal *P_heightscale_*, *P_timescale_* and also the prepubertal *Q_heightscale_*. For determining, for instance, the location of *AgeT_PHV_*, we first look at the location of typical mean *mAgeT_PHV_* at the zero point of the typical mean height acceleration function *mT''(mAgeT_PHV_)*= *mP''(mAgeT_PHV_)*+2*Q_p2_*=0. For the location of the individual *AgeT_PHV_*, the zero point of individual HA-function is *T''(AgeT_PHV_)*= *P''(AgeT_PHV_)*+*Q''(AgeT_PHV_)*= (*P_heightscale_ /P_timescale_^2^)mP''(mAgeP50* + *(AgeT_PHV_* –*AgeP50)*/ *P_timescale_)* + *Q_heightscale_* 2*Q_p2_*=0. Focussing on quantifying the shift of *AgeT_PHV_* compared to mid-puberty, the equation can be rewritten via *mP''(mAgeP50 +* *(AgeT_PHV_* – *AgeP50)/P_timescale_)* = –*P_timescale_^2^ Q_heightscale_* 2*Q_p2_*/ *P_heightscale_* into *(AgeT_PHV_* –*AgeP50)* = *P_timescale_ (mP''^inverse^(*–*P_timescale_^2^ Q_heightscale_* 2*Q_p2_*/ *P_heightscale_)* –*mAgeP50)*, showing that this individual shift is a nonlinear function of the variables *P_heightscale_*, *P_timescale_* and *Q_heightscale_*, independent of age by inverting function *mP''(age)* between *AgeT_PHA_* and *AgeP50*. By inverting *mP''(age)* below *AgeT_PHA_* also the shift of *AgeT_ONSET_* compared to *AgeP50* can be exactly computed.

In Figure A4 we illustrate the shift of *AgeT_PHV_* compared to mid-puberty for a girl with low pubertal growth spurt, *P_heightscale_* =0.4 and high prepubertal *Q_heightscale_* =1.2. Compared with Figure 2, it can be observed that, if the maximum value of HA *T''(age)* decreases, *AgeT_ONSET_* and *AgeT_PHV_* shift closer to each other, until finally they cannot be estimated anymore when the maximum HA (*T_PHA_*, *T* peak height acceleration) value at *AgeT_PHA_* drops below zero. The resulting three conditions are: if *T_PHA_* < 0, then *AgeT_ONSET_* and *AgeT_PHV_* cannot be estimated, if *T_PHA_* = 0, then *AgeT_PHA_* = *AgeT_ONSET_* = *AgeT_PHV_*, and if *T_PHA_* > 0, then *AgeT_ONSET_* < *AgeT_PHA_* < *AgeT_PHV_* < *AgeP50* or in other words if *T_PHA_* is positive *AgeT_PHV_* is always between *AgeT_PHA_* and *AgeP50*. Notice that the age at peak HA of *T*-function and *P*-function are always equal, *AgeT_PHA_* = *AgeP_PHA_* both for boys and girls at the age when 19% of *P*-function growth is reached.

**A1:3 *PQ_ratio_*, related to age expressed in percentage of the *P-*function**

*PQ_ratio_* is defined here as the overall HA ratio of *P_max_* divided by *Q_max_* rescaled in such a way that the expected value is 1 for an individual having QEPS typical mean growth:

*PQ_ratio_ = (P_heightscale_ /P_timescale_^2^) / Q_heightscale_*.

The HA ratio *PQ_ratio_* can be used to assess the relative influence of *Q* and *P* on the age of puberty onset *AgeT_ONSET_* and the age of PHV *AgeT_PHV_* of the *T*-function. Age as *AgeT_XXX_* can be expressed in percentage of the *P-*function by computing *P_perc_(AgeT_XXX_)* = 100**P(AgeT_XXX_)/P_max_* with the individual *P-f*unction or as well by computing *P_perc_(AgeT_XXX_)* = 100**mP(mAgeP50 +* *(AgeT_XXX_* – *AgeP50)/P_timescale_)/mP_max_* with the typical mean *mP*-function, giving exactly the same percentage. By inverting *mP* we can rewrite this equation in *mP^inverse^*(*P_perc_(AgeT_XXX_) mP_max_/*100) = *mAgeP50 +* *(AgeT_XXX_* – *AgeP50)/P_timescale_* and substitute together with *PQ_ratio_* in the formula of the previous subchapter *mP''(mAgeP50 +* *(AgeT_PHV_* – *AgeP50)/P_timescale_)* = –*P_timescale_^2^ Q_heightscale_* 2*Q_p2_*/ *P_heightscale_* giving *PQ_ratio_*mP''(mP^inverse^*(*P_perc_(AgeT_XXX_) mP_max_/*100)*)* = –2*Q_p2_*, showing that each individual value of *PQ_ratio_* corresponds to a functional depending value of *AgeT_XXX_*, in this case of puberty onset *AgeT_ONSET_* and PHV *AgeT_PHV_*. Individual values of the population for *P_perc_* at *AgeT_ONSET_*, *AgeT_PHV_* and *AgeT_END_* are shown in Figure A2, at the left for *AgeT_ONSET_*, in the middle for *AgeT_PHV_* and at the right for *AgeT_END_*. *P_perc_(AgeT_ONSET_)* and *P_perc_(AgeT_PHV_)* clearly show the functional relation with *PQ_ratio_*, whereas *P_perc_(AgeT_END_)* shows extra variation introduced by the influence of the *S-*function.

**A.2 *MathSelect***

**A.2:1 Construction of *MathSelect***

The mathematical selection criterion *MathSelect* combines nine input variables for assessing the quality of the fitted total individual height function *T(age)*: *T_SDerror_* (the standard error of the residuals), *AgeP50_CI_, P_heightscaleCI_, P_timescaleCI_, SP_heightinterceptCI_, SP_heightscaleCI_, E_timescaleCI_* (respectively half of the estimated 95% confidence interval of *AgeP50, P_heightscale_, P_timescale_, SP_heightintercept_, SP_heightscale_* and *E_timescale_*), *ΔTmaxAH* (maximum observed adult height minus modelled adult height, *AH* – *T_max_*) and *Penalty*, giving the specific logit transformed penalty ratio *Logit(PenRatio)* = log10((0.001+0.998**PenRatio*)/(0.999–0.998**PenRatio*))*,* with penalty ratio *PenRatio* = *PEN* / *PENSSE*, with penalty function *PEN* and penalized residual sum of squares *PENSSE* as defined in Nierop et al. 2016, appendix A.9.

*MathSelect* was constructed in three steps:

1. In the first step, empirical cumulative distributions of the nine input variables were fitted for boys and girls with 16 parametric cumulative distribution functions (CDFs). In the selection process for suitable candidates we aimed at limiting the number of different distributions and the number of fitted parameters as much as possible. Finally, fitting two parameters appeared to be sufficient for all, with the loglogistic distribution as most appropriate for *T_SDerror_* and the six *CI* variables, the logistic distribution for *ΔTmaxAH* and the normal distribution for *Penalty*. The selected distributions for the input CDFs are summarized in Table A4, with corresponding parameter estimates for *F(Input* | *mu,sigma)*.

2. In the second *MathSelect* step, the individual maximum cumulative probability (*MaxCDF*) was selected from the nine input parametric CDF estimates, where the cumulative probabilities of the *Logistic(ΔTmaxAH)* CDFs were transformed for girls and boys to

(*Logistic(ΔTmaxAH)* –0.5) to monitor the percentiles of this input variable two-sided around the median. In formula *MaxCDF* is computed as: *MaxCDF* = max(*F_TSDerror_*(*T_SDerror_*), *F_AgeP50CI_*(*AgeP50_CI_*), *F_PheightscaleCI_*(*P_heightscaleCI_*), *F_PtimescaleCI_*(*P_timescaleCI_*), *F_SPheightinterceptCI_*(*SP_heightinterceptCI_*), *F_SPheightscaleCI_*(*SP_heightscaleCI_*), *F_EtimescaleCI_*(*E_timescaleCI_*), 2*abs(*F_ΔTmaxAH_*(*ΔTmaxAH*) –0.5), *F_Penalty_*(*Penalty*))

3. In the third *MathSelect* step the empirical cumulative distributions of *MaxCDF* were fitted for boys and girls with 16 CDFs and the Beta CDF selected as most appropriate, with corresponding parameter estimates for *F(MaxCDF* | *a,b)* = *F_MaxCDF_*(*MaxCDF*) at the bottom of Table A4.

**A.2:2 Functional boundaries for *MathSelect* input variables**

For each of the nine input variables used for constructing *MathSelect*, extreme possible values can be computed by back transforming *MathSelect* values using quantile functions (inverse CDFs). Also the order of the three steps for constructing *MathSelect* has to be reversed. Reversing step three gives quantile function *Q_MaxCDF_*(*MathSelect*), the inverse of *F_MaxCDF_*(*MaxCDF*), and consecutively reversing step one gives quantile function *Q_Input_*(*Q_MaxCDF_*(*MathSelect*)), the inverse of *F_Input_*(*Input*). The input quantile function *Q_Input_*(*Q_MaxCDF_*(*MathSelect*)) gives upper boundaries for each value of *MathSelect*, because in the second *MathSelect* step the individual maximum cumulative probability has been selected. Only quantile function *Q_ΔTmaxAH_* will give upper and lower boundaries: due to the transformation of the cumulative probabilities of the *Logistic(ΔTmaxAH)* CDFs in the second step, *Q_MaxCDF_*(*MathSelect*) needs to be transformed for *Q_ΔTmaxAH_* as *Q_ΔTmaxAHupper_*(0.5+0.5**Q_MaxCDF_*(*MathSelect*)) for the upper and *Q_ΔTmaxAHlower_*(0.5–0.5**Q_MaxCDF_*(*MathSelect*)) for the lower bound.

Legends to: Additional Figures

**Figure A1. Illustration of growth from birth to adult height separated into all QEPS growth functions, of the four individuals of Figure 3.**

The total growth in height of the four individuals in Figure 3 are shown with the growth functions related to chronological years, as well as relative pubertal age, for comparison.

**For each of the four individuals: to the left,** are the *Q, E*, and *QE*-functions shown in both cm, and SD-scores (height_SDS_), upper part until 18 years, lower part until 5 years of age. **To the right** are the *P*-, *QES*- and *QEPS* (=*T*, total height) -functions shown, the upper part shown in chronological years and the lower part in age from individualized onset of puberty as shown in Figure 3 of the main manuscript**.** The upper solid black lines indicate the individual *QE-*function expressed in cm and height_SDS,_ in the left panels and *T-*function in cm and height_SDS_, in the right panels with the actual measurements indicated as circles. The gray solid line indicate the mean *QE-*function of the study population in the left panels, the mean *T-*function of the study population in the right panels, here reference, and the dotted gray lines represent ± 2 SD-scores of the reference. The solid blue line in the right panels shows the individual height *QES*-function expressed in cm and height_SDS_, the solid light blue line the mean *QES* values, and the dotted light blue lines ± 2 SD-scores of the reference. The solid green line shows the individual height *E*-function expressed in cm and height_SDS_, the solid light green line the mean *E* values, and the dotted light green lines ± 2 SD-scores of the reference. The solid red line shows the individual specific *P-*function expressed in cm and height_SDS_, the solid light red line showing the mean *P-*values, and the dotted light red lines represent ±2 SD.

**Figure A1.A** shows a girl with a low and decelerating *QE*-function with height_SDS_ from -2 to -2.5 during childhood, with a late but high *P-*function height_SDS_ +1.2, resulting in an adult height of -1.7. **Figure A1.B** shows a girl with early and low *P-*function height_SDS_ compensated by high *QES-*function height_SDS_ around +1.5/ 2.0. The resulting *T-*function height_SDS_ is as highest between 8-10 years of age,(approx +1.8, but due to both a declining *QES-*function from 11 years of age and a weak *P-*function is her adult height_SDS_ ending up around +0.4. **Figure A1.C** shows a boy with declining *QES*-function height_SDS_, entering puberty late, with a high *P*-function height_SDS_ of +1.8 and ending up with an adult height_SDS_ of -0.1. **Figure A1.D** shows a boy with a high and accelerating *QES*-function height_SDS_ above +3.0 at the end of puberty; he enters puberty early but in the end is the *P*-function height_SDS_ -0.7, resulting in *T*-function adult height_SDS_ of +1.7 /200 cm.

**Figure A2. Scatter plot showing the relationship between *PQ_ratio_* and percentage of the *P*-function for *AgeT_ONSET_*, *AgeT_PHV_* and *AgeT_END_*.**

The girls are shown as red circles and the boys as blue crosses. Assuming that the percentage of the *P*-function is giving the best estimated individual puberty age, this scatter plot shows how the estimation of puberty age with the *T*-function is systematically influenced by *PQ_ratio_*, the HA ratio of the individual *P* and *Q* height-scale parameters. At the left in the figure, individual onset of pubertal growth, *AgeT_ONSET_*, is transformed in corresponding individual best estimated puberty age and plotted versus the *PQ_ratio_*. The median value of *AgeT_ONSET_* is 2% for girls and 1% for boys. Assuming that the median onset of pubertal growth estimated with the *T*-function is the expected age of onset for this group, it can be seen that a relative low *P_heightscale_* compared to *Q_heightscale_* systematically gives a later estimation for *AgeT_ONSET_*, a shorter duration from *AgeT_ONSET_* to *AgeT_END_* at the right and a systematic lower *TpubTgain*, since this pubertal gain due to *T*-function growth is computed with these two variables. The median value of *AgeT_END_* is 97% for girls and boys. Also the estimation of *AgeT_PHV_* in the middle of the figure is influenced by the *PQ_ratio_*. The median value of *AgeT_PHV_*, age at PHV of the *T-*function, is 43% for girls and 45% for boys. Assuming that the median *AgeT_PHV_* is the expected age at PHV for this group when only total height measurements are evaluated, it can be seen that a relative low *P_heightscale_* compared to *Q_heightscale_* systematically gives an earlier estimation for *AgeT_PHV_* and vice versa. Especially for girls the estimation of puberty age parameters with the *T*-function is considerably influenced by the individual *P* and *Q* height-scale parameters.

**Figure A3. Scatter plot showing the relationship between adult height and onset of pubertal growth (*AgeT_ONSET_*)**

Age at the minimum height velocity before the pubertal growth spurt (*AgeT_ONSET_*) for girls (red circles) and boys (blue crosses) in the study population is related to adult height. For girls; adult height = 156.64 + 1.189 x *AgeT_ONSET_*, adjusted r^2^ = 0.0376 (i.e. a 1-year delay will give a 1.19 cm taller adult height). For boys; adult height = 171.87+ 0.818 x *AgeT_ONSET_*, adjusted r^2^ =0.0131 (i.e. a 1-year delay will give a 0.82 cm taller adult height).

**Figure A4. Height velocity graph with QEPS puberty estimates** **showing a girl with low pubertal gain.**

The individual pubertal QEPS parameters are shown to be influenced by decreasing the pubertal growth spurt. The shadowed area, *P_max_* (total *P-*function growth) is small, giving insecurity in the location of *AgeT_PHV_* and *AgeT_ONSE_*_T_, corresponding to high CIs for these pubertal growth estimates. If there is low pubertal height gain, the height acceleration curve *T’’(age)* will no longer cross the horizontal zero line, hence *AgeT_PHV_* and *AgeT_ONSET_,* cannot be estimated.

**Figure A5. Scatterplot showing the relationship between *individual P_max_***–***P_maxCI_* and *individual P_max._***

The relationship between the pubertal gain as *P_max_* (the total *P-*function growth) minus half of the corresponding estimated 95% confidence interval (*P_maxCI_*) is related to *P_max_,* for girls (red circles) and boys (blue crosses) in the study population. When *P_max_* decreases it finally reaches a level where it cannot be found above the 95% confidence limit; for girls; 3.3 cm for boys; 2.8 cm.

**Tables Additional file**

**Table A1A Age in years for pubertal growth estimates in girls**

| **Variable** | **N** | **Mean** | **Median** | **SD** | **Max** | **Min** |
| --- | --- | --- | --- | --- | --- | --- |
| **Onset of puberty** |  |  |  |  |  |  |
| **AgeTonset**, age at minimum height velocity of the *T*-function^1^ | 1129 | 9.24 | 9.19 | 1.01 | 12.62 | 6.37 |
| **AgeP1**, age at 1% of the *P*-function^2^ | 1139 | 8.71 | 8.68 | 0.98 | 12.00 | 6.09 |
| **AgeP5**, age at 5% of the *P*-function | 1139 | 9.86 | 9.81 | 0.97 | 13.13 | 7.30 |
| **Mid puberty** |  |  |  |  |  |  |
| **AgePHV**, age at visual estimated PHV | 1134 | 11.92 | 11.88 | 0.97 | 15.31 | 9.35 |
| **AgeTPHV**, age at PHV of the *T*-function | 1129 | 11.83 | 11.80 | 0.96 | 15.09 | 9.39 |
| **AgePPHV**, age at PHV of the *P*-function | 1139 | 12.02 | 11.98 | 0.95 | 15.26 | 9.51 |
| **AgeP50**, age at 50% of the *P*-function | 1139 | 12.09 | 12.06 | 0.95 | 15.34 | 9.59 |
| **End of puberty** |  |  |  |  |  |  |
| **AgeP95**, age at 95% of the *P*-function | 1139 | 14.66 | 14.65 | 0.95 | 17.93 | 12.23 |
| **AgeP99**, age at 99% of the *P*-function | 1139 | 16.33 | 16.34 | 0.95 | 19.63 | 13.91 |
| **AgeTend**, age where the height velocity has decreased to 1 cm/year | 1139 | 15.01 | 15.03 | 0.84 | 18.00 | 12.85 |
| **Differences** |  |  |  |  |  |  |
| Difference AgePHV – AgeTPHV | 1125 | 0.09 | 0.08 | 0.45 | 2.27 | -1.41 |
| Difference AgeP50 – AgePHV | 1134 | 0.17 | 0.18 | 0.46 | 1.98 | -1.98 |
| Difference AgeP50 – AgeTPHV | 1129 | 0.26 | 0.24 | 0.08 | 0.93 | 0.10 |
| Difference AgePPHV – AgePHV | 1134 | 0.10 | 0.11 | 0.46 | 1.90 | -2.06 |
| Difference AgeP50 – AgePPHV | 1139 | 0.08 | 0.08 | 0.00 | 0.09 | 0.05 |
| Difference AgePPHV – AgeTPHV | 1129 | 0.18 | 0.16 | 0.08 | 0.85 | 0.05 |
| **Duration** |  |  |  |  |  |  |
| **Duration between AgeP5 and AgeP95** | 1139 | 4.80 | 4.78 | 0.21 | 5.54 | 3.44 |
| Duration between AgeP5 and AgeP50 | 1139 | 2.23 | 2.22 | 0.10 | 2.57 | 1.60 |
| Duration between AgeP50 and AgeP95 | 1139 | 2.57 | 2.56 | 0.11 | 2.96 | 1.84 |
| Duration between AgeP1 and AgeP99 | 1139 | 7.61 | 7.58 | 0.33 | 8.77 | 5.46 |
| Duration between AgeP1 and AgeP50 | 1139 | 3.38 | 3.36 | 0.15 | 3.89 | 2.42 |
| Duration between AgeP50 and AgeP99 | 1139 | 4.24 | 4.22 | 0.18 | 4.88 | 3.04 |
| Duration between AgeTonset and AgeTPHV | 1129 | 2.59 | 2.65 | 0.39 | 3.53 | 0.43 |
| Duration between AgeTonset and AgeTend | 1129 | 5.77 | 5.78 | 0.50 | 7.11 | 3.84 |

^1^Total height function in cm; *T(age) = Q(age) + E(age) + P(age) – S(age), ^2^*Quadratic logistic function describing the pubertal growth spurt *P(age)* in cm.

**Table A1B Age in years for pubertal growth estimates in boys**

| **Variable** | **N** | **Mean** | **Median** | **SD** | **Max** | **Min** |
| --- | --- | --- | --- | --- | --- | --- |

| **Onset of puberty** | |  |  |  |  |  |
| --- | --- | --- | --- | --- | --- | --- |
| **AgeTonset**, age at minimum height velocity of the *T*-function^1^ | 1141 | 10.74 | 10.71 | 0.98 | 14.20 | 7.50 |
| **AgeP1**, age at 1% of the *P*-function^2^ | 1141 | 10.73 | 10.72 | 0.97 | 13.94 | 7.45 |
| **AgeP5**, age at 5% of the *P*-function | 1141 | 11.78 | 11.77 | 0.96 | 14.98 | 8.56 |
| **Mid puberty** | |  |  |  |  |  |
| **AgePHV**, age at visual estimated PHV | 1136 | 13.83 | 13.81 | 1.00 | 17.18 | 10.95 |
| **AgeTPHV**, age at PHV of the *T*-function | 1141 | 13.66 | 13.65 | 0.96 | 16.84 | 10.54 |
| **AgePPHV**, age at PHV of the *P*-function | 1141 | 13.73 | 13.72 | 0.96 | 16.94 | 10.63 |
| **AgeP50**, age at 50% of the *P*-function | 1141 | 13.80 | 13.78 | 0.96 | 17.01 | 10.69 |
| **End of puberty** | |  |  |  |  |  |
| **AgeP95**, age at 95% of the *P*-function | 1141 | 16.10 | 16.06 | 0.97 | 19.31 | 13.12 |
| **AgeP99**, age at 99% of the *P*-function | 1141 | 17.56 | 17.52 | 0.98 | 20.78 | 14.58 |
| **AgeTend**, age where the height velocity has decreased to 1 cm/year | 1141 | 16.68 | 16.64 | 0.90 | 19.44 | 14.01 |

| **Differences** |  |  |  |  | |  | |  |
| --- | --- | --- | --- | --- | --- | --- | --- | --- |
| Difference AgePHV – AgeTPHV | 1136 | 0.18 | 0.16 | | 0.35 | | 1.40 | -0.83 |
| Difference AgeP50 – AgePHV | 1136 | -0.04 | -0.02 | | 0.36 | | 0.97 | -1.26 |
| Difference AgeP50 – AgeTPHV | 1141 | 0.14 | 0.14 | | 0.03 | | 0.41 | 0.08 |
| Difference AgePPHV – AgePHV | 1136 | -0.10 | -0.08 | | 0.36 | | 0.90 | -1.32 |
| Difference AgeP50 – AgePPHV | 1141 | 0.06 | 0.06 | | 0.00 | | 0.08 | 0.05 |
| Difference AgePPHV – AgeTPHV | 1141 | 0.08 | 0.07 | | 0.02 | | 0.35 | 0.03 |

| **Duration** |  | |  |  |  |  |  |
| --- | --- | --- | --- | --- | --- | --- | --- |
| **Duration between AgeP5 and AgeP95** | | 1141 | 4.32 | 4.31 | 0.22 | 5.55 | 3.24 |
| Duration between AgeP5 and AgeP50 | | 1141 | 2.02 | 2.02 | 0.10 | 2.60 | 1.51 |
| Duration between AgeP50 and AgeP95 | | 1141 | 2.30 | 2.30 | 0.12 | 2.96 | 1.72 |
| Duration between AgeP1 and AgeP99 | | 1141 | 6.83 | 6.82 | 0.34 | 8.78 | 5.12 |
| Duration between AgeP1 and AgeP50 | | 1141 | 3.06 | 3.06 | 0.15 | 3.94 | 2.30 |
| Duration between AgeP50 and AgeP99 | | 1141 | 3.77 | 3.76 | 0.19 | 4.84 | 2.82 |
| Duration between AgeTonset and AgeTPHV | | 1141 | 2.92 | 2.93 | 0.25 | 3.74 | 1.49 |
| Duration between AgeTonset and AgeTend | | 1141 | 5.94 | 5.94 | 0.38 | 7.62 | 4.37 |

^1^Total height function in cm; *T(age) = Q(age) + E(age) + P(age) – S(age), ^2^*Quadratic logistic function describing the pubertal growth spurt *P(age)* in cm.

**Table A2A Estimated heights and pubertal gains in cm for girls**

| **Variable** | **N** | **Mean** | **Median** | **SD** | **Max** | **Min** |
| --- | --- | --- | --- | --- | --- | --- |
| **Onset of puberty, heights at;** | |  |  |  |  |  |
| **AgeTonset**, age at minimum height velocity of the *T*-function^1^ | 1129 | 136.15 | 135.87 | 7.78 | 160.45 | 111.52 |
| **AgeP1**, age at 1% of the *P*-function^2^ | 1139 | 133.34 | 133.34 | 7.09 | 155.41 | 110.94 |
| **AgeP5**, age at 5% of the *P*-function | 1139 | 139.47 | 139.36 | 6.91 | 160.72 | 116.45 |
| **Mid puberty, heights at;** | |  |  |  |  |  |
| **AgeTPHV**, age at peak height velocity from the *T*-function | 1129 | 152.37 | 152.32 | 6.15 | 169.62 | 130.45 |
| **AgeP50**, age at 50% of the *P*-function | 1139 | 154.29 | 154.07 | 6.25 | 171.68 | 131.92 |
| **End of puberty, heights at;** | |  |  |  |  |  |
| **AgeP95**, age at 95% of the *P*-function | 1139 | 165.81 | 165.77 | 6.04 | 181.91 | 144.26 |
| **AgeP99**, age at 99% of the *P*-function | 1139 | 166.98 | 167.02 | 6.04 | 183.09 | 145.41 |
| **AgeTend**, age where the height velocity has decreased to 1 cm/year | 1139 | 166.24 | 166.24 | 6.05 | 182.39 | 144.65 |
| Adult height | 1139 | 167.66 | 167.60 | 6.06 | 183.7 | 146.5 |
| **Height gains in cm between;** | |  |  |  |  |  |
| AgeP5 and AgeP95 | 1139 | 26.34 | 26.35 | 3.81 | 37.86 | 12.98 |
| AgeP5 and AgeP50 | 1139 | 14.82 | 14.81 | 2.00 | 20.76 | 8.13 |
| AgeP50 and AgeP95 | 1139 | 11.52 | 11.51 | 1.82 | 17.10 | 4.85 |
| AgeP1 and AgeP99 | 1139 | 33.64 | 33.57 | 4.56 | 47.33 | 18.62 |
| AgeTonset and AgeTend | 1129 | 30.09 | 30.14 | 5.18 | 45.56 | 11.79 |
| AgeTonset and AgeTPHV | 1129 | 13.87 | 13.84 | 2.01 | 20.04 | 7.71 |
| AgeTPHV and AgeTend | 1129 | 16.23 | 16.33 | 3.35 | 25.53 | 2.54 |
| AgeTonset and adult height | 1129 | 31.51 | 31.54 | 5.34 | 46.86 | 12.73 |

^1^Total height function in cm; *T(age) = Q(age) + E(age) + P(age) – S(age), ^2^*Quadratic logistic function describing the pubertal growth spurt *P(age)* in cm.

**Table A2B Estimated heights and pubertal gains in cm for boys**

| **Variable** | **N** | **Mean** | **Median** | **SD** | **Max** | **Min** |
| --- | --- | --- | --- | --- | --- | --- |
| **Onset of puberty, heights at;** | |  |  |  |  |  |
| **AgeTonset**, age at minimum height velocity of the *T*-function^1^ | 1141 | 144.60 | 144.28 | 7.45 | 168.17 | 116.45 |
| **AgeP1**, age at 1% of the *P*-function^2^ | 1141 | 144.53 | 144.17 | 7.03 | 166.56 | 117.83 |
| **AgeP5**, age at 5% of the *P*-function | 1141 | 149.76 | 149.36 | 6.97 | 170.80 | 122.56 |
| **Mid puberty, heights at;** | |  |  |  |  |  |
| **AgeTPHV**, age at peak height velocity from the *T*-function | 1141 | 163.74 | 163.40 | 6.54 | 182.12 | 138.82 |
| **AgeP50**, age at 50% of the *P*-function | 1141 | 165.03 | 164.67 | 6.58 | 183.59 | 140.06 |
| **End of puberty, heights at;** | |  |  |  |  |  |
| **AgeP95**, age at 95% of the *P*-function | 1141 | 178.75 | 178.50 | 6.59 | 199.06 | 155.64 |
| **AgeP99**, age at 99% of the *P*-function | 1141 | 180.15 | 179.85 | 6.62 | 200.77 | 157.01 |
| **AgeTend**, age where the height velocity has decreased to 1 cm/year | 1141 | 179.62 | 179.35 | 6.62 | 200.29 | 156.49 |
| Adult height | 1141 | 180.69 | 180.40 | 6.63 | 201.7 | 157.3 |
| **Height gains in cm between;** | |  |  |  |  |  |
| AgeP5 and AgeP95 | 1141 | 29.00 | 28.97 | 3.64 | 40.17 | 16.68 |
| AgeP5 and AgeP50 | 1141 | 15.27 | 15.27 | 1.84 | 21.31 | 9.17 |
| AgeP50 and AgeP95 | 1141 | 13.73 | 13.73 | 1.80 | 18.90 | 7.51 |
| AgeP1 and AgeP99 | 1141 | 35.62 | 35.55 | 4.26 | 49.39 | 22.17 |
| AgeTonset and AgeTend | 1141 | 35.02 | 35.08 | 4.74 | 48.61 | 16.41 |
| AgeTonset and AgeTPHV | 1141 | 15.88 | 15.88 | 2.07 | 22.15 | 9.54 |
| AgeTPHV and AgeTend | 1141 | 19.14 | 19.18 | 2.70 | 26.46 | 6.80 |
| AgeTonset and adult height | 1141 | 36.09 | 36.10 | 4.89 | 51.39 | 17.16 |

^1^Total height function in cm; *T(age) = Q(age) + E(age) + P(age) – S(age), ^2^*Quadratic logistic function describing the pubertal growth spurt *P(age)* in cm.

**Table A3A The *MathSelect* (MS) function for subgroup selection. Study group, no MS**

| **Girls** | | | | | | | | | | |
| --- | --- | --- | --- | --- | --- | --- | --- | --- | --- | --- |
| **Variable** | **N** | **Mean** | **Med-an** | **SD** | **Skew-ness** | **Kurto-sis** | **Max** | **Min** | **Lower 95%** | **Upper 95%** |
|  |  |  |  |  |  |  |  |  | **CL for Mean** | **CL for Mean** |
| AgeTonset^1^ | 1129 | 9.24 | 9.19 | 1.01 | 0.18 | 0.02 | 12.62 | 6.37 | 9.19 | 9.30 |
| AgeP5^2^ | 1139 | 9.86 | 9.81 | 0.97 | 0.15 | 0.04 | 13.13 | 7.30 | 9.80 | 9.92 |
| AgeTPHV^3^ | 1129 | 11.83 | 11.80 | 0.96 | 0.14 | 0.02 | 15.09 | 9.39 | 11.78 | 11.89 |
| AgePPHV^4^ | 1139 | 12.02 | 11.98 | 0.95 | 0.15 | 0.015 | 15.26 | 9.51 | 11.96 | 12.07 |
| AgeP50^5^ | 1139 | 12.09 | 12.06 | 0.95 | 0.15 | 0.014 | 15.34 | 9.59 | 12.04 | 12.15 |
| AgeP95^6^ | 1139 | 14.66 | 14.65 | 0.95 | 0.14 | -0.022 | 17.93 | 12.23 | 14.61 | 14.72 |
| AgeTonsetCI | 1139 | 0.87 | 0.77 | 0.49 | 6.42 | 73.64 | 8.37 | 0.22 | 0.84 | 0.89 |
| AgeP5CI | 1139 | 0.93 | 0.81 | 0.54 | 6.70 | 94.17 | 10.51 | 0.21 | 0.90 | 0.96 |
| AgeTPHVCI | 1139 | 0.63 | 0.54 | 0.45 | 8.24 | 114.47 | 8.37 | 0.14 | 0.61 | 0.66 |
| AgePPHVCI | 1139 | 0.57 | 0.51 | 0.32 | 7.06 | 103.03 | 6.42 | 0.14 | 0.55 | 0.59 |
| AgeP50CI | 1139 | 0.56 | 0.51 | 0.32 | 6.99 | 101.13 | 6.28 | 0.13 | 0.55 | 0.58 |
| AgeP95CI | 1139 | 0.70 | 0.64 | 0.31 | 2.92 | 16.67 | 3.42 | 0.16 | 0.69 | 0.72 |
| **Boys** | | | | | | | | | | |
| **Variable** | **N** | **Mean** | **Medi-an** | **SD** | **Skew-ness** | **Kurto-sis** | **Max** | **Min** | **Lower 95%** | **Upper 95%** |
|  |  |  |  |  |  |  |  |  | **CL for Mean** | **CL for Mean** |
| AgeTonset^1^ | 1141 | 10.74 | 10.71 | 0.98 | 0.15 | 0.11 | 14.20 | 7.50 | 10.68 | 10.80 |
| AgeP5^2^ | 1141 | 11.78 | 11.77 | 0.96 | 0.13 | 0.013 | 14.98 | 8.56 | 11.72 | 11.83 |
| AgeTPHV^3^ | 1141 | 13.66 | 13.65 | 0.96 | 0.14 | 0.017 | 16.84 | 10.54 | 13.60 | 13.71 |
| AgePPHV^4^ | 1141 | 13.73 | 13.72 | 0.96 | 0.15 | 0.030 | 16.94 | 10.63 | 13.68 | 13.79 |
| AgeP50^5^ | 1141 | 13.80 | 13.78 | 0.96 | 0.15 | 0.030 | 17.01 | 10.69 | 13.74 | 13.85 |
| AgeP95^6^ | 1141 | 16.10 | 16.06 | 0.97 | 0.15 | 0.034 | 19.31 | 13.12 | 16.04 | 16.15 |
| AgeTonsetCI | 1141 | 0.73 | 0.66 | 0.31 | 3.72 | 33.80 | 4.86 | 0.28 | 0.71 | 0.74 |
| AgeP5CI | 1141 | 0.75 | 0.67 | 0.36 | 4.26 | 42.15 | 5.86 | 0.27 | 0.73 | 0.77 |
| AgeTPHVCI | 1141 | 0.46 | 0.41 | 0.22 | 4.22 | 42.34 | 3.53 | 0.11 | 0.45 | 0.47 |
| AgePPHVCI | 1141 | 0.44 | 0.40 | 0.19 | 3.59 | 32.34 | 3.03 | 0.11 | 0.43 | 0.45 |
| AgeP50CI | 1141 | 0.44 | 0.40 | 0.19 | 3.48 | 30.32 | 2.94 | 0.11 | 0.43 | 0.45 |
| AgeP95CI | 1141 | 0.62 | 0.55 | 0.27 | 2.74 | 13.16 | 2.72 | 0.22 | 0.60 | 0.64 |

Pubertal growth estimates. ^1^Age at minimum height velocity of *T*, the total height function, from the QEPS-model. ^2^Age at 5% of *P*, the pubertal growth function, from the QEPS-model. ^3^Age at peak height velocity from the *T*-function. ^4^Age at peak height velocity from the *P-*function. ^5^Age at 50% of the *P-*function. ^6^Age at 95% of the *P-*function. CI = half of the estimated 95% confidence interval in years for the pubertal growth estimates.

**Table A3B The *MathSelect* (MS) function for subgroup selection. MS** **<0.975**

| **Girls** | | | | | | | | | | |
| --- | --- | --- | --- | --- | --- | --- | --- | --- | --- | --- |
| **Variable** | **N** | **Mean** | **Medi-an** | **SD** | **Skew-ness** | **Kurto-sis** | **Max** | **Min** | **Lower 95%** | **Upper 95%** |
|  |  |  |  |  |  |  |  |  | **CL for Mean** | **CL for Mean** |
| AgeTonset^1^ | 1110 | 9.24 | 9.19 | 1.00 | 0.15 | -0.03 | 12.58 | 6.37 | 9.18 | 9.29 |
| AgeP5^2^ | 1116 | 9.85 | 9.81 | 0.96 | 0.11 | -0.02 | 13.03 | 7.30 | 9.79 | 9.91 |
| AgeTPHV^3^ | 1110 | 11.83 | 11.81 | 0.96 | 0.10 | -0.06 | 15.09 | 9.39 | 11.77 | 11.88 |
| AgePPHV^4^ | 1116 | 12.01 | 11.98 | 0.95 | 0.11 | -0.06 | 15.23 | 9.51 | 11.95 | 12.06 |
| AgeP50^5^ | 1116 | 12.08 | 12.05 | 0.95 | 0.11 | -0.06 | 15.31 | 9.59 | 12.03 | 12.14 |
| AgeP95^6^ | 1116 | 14.66 | 14.65 | 0.94 | 0.10 | -0.10 | 17.93 | 12.23 | 14.60 | 14.71 |
| AgeTonsetCI | 1116 | 0.83 | 0.76 | 0.33 | 2.23 | 10.51 | 3.52 | 0.22 | 0.81 | 0.85 |
| AgeP5CI | 1116 | 0.89 | 0.80 | 0.37 | 1.61 | 3.64 | 2.86 | 0.21 | 0.87 | 0.91 |
| AgeTPHVCI | 1116 | 0.60 | 0.54 | 0.28 | 2.27 | 8.40 | 2.67 | 0.14 | 0.58 | 0.62 |
| AgePPHVCI | 1116 | 0.55 | 0.51 | 0.22 | 1.51 | 3.73 | 1.73 | 0.14 | 0.54 | 0.56 |
| AgeP50CI | 1116 | 0.54 | 0.50 | 0.22 | 1.52 | 3.78 | 1.72 | 0.13 | 0.53 | 0.55 |
| AgeP95CI | 1116 | 0.68 | 0.63 | 0.25 | 1.16 | 1.72 | 1.73 | 0.16 | 0.67 | 0.70 |
| **Boys** | | | | | | | | | | |
| **Variable** | **N** | **Mean** | **Medi-an** | **SD** | **Skew-ness** | **Kurto-sis** | **Max** | **Min** | **Lower 95%** | **Upper 95%** |
|  |  |  |  |  |  |  |  |  | **CL for Mean** | **CL for Mean** |
| AgeTonset^1^ | 1115 | 10.72 | 10.71 | 0.96 | 0.12 | 0.062 | 13.81 | 7.50 | 10.67 | 10.78 |
| AgeP5^2^ | 1115 | 11.77 | 11.77 | 0.95 | 0.097 | -0.015 | 14.70 | 8.56 | 11.71 | 11.82 |
| AgeTPHV^3^ | 1115 | 13.64 | 13.64 | 0.95 | 0.11 | -0.014 | 16.63 | 10.54 | 13.59 | 13.70 |
| AgePPHV^4^ | 1115 | 13.72 | 13.71 | 0.95 | 0.12 | -0.005 | 16.73 | 10.63 | 13.67 | 13.78 |
| AgeP50^5^ | 1115 | 13.78 | 13.78 | 0.94 | 0.12 | -0.005 | 16.79 | 10.69 | 13.73 | 13.84 |
| AgeP95^6^ | 1115 | 16.08 | 16.06 | 0.95 | 0.13 | -0.005 | 19.17 | 13.12 | 16.03 | 16.14 |
| AgeTonsetCI | 1115 | 0.71 | 0.66 | 0.24 | 1.31 | 2.41 | 2.05 | 0.28 | 0.69 | 0.72 |
| AgeP5CI | 1115 | 0.73 | 0.67 | 0.28 | 1.58 | 3.66 | 2.21 | 0.27 | 0.71 | 0.74 |
| AgeTPHVCI | 1115 | 0.45 | 0.41 | 0.17 | 1.57 | 4.72 | 1.75 | 0.11 | 0.44 | 0.46 |
| AgePPHVCI | 1115 | 0.43 | 0.39 | 0.16 | 1.21 | 1.88 | 1.13 | 0.11 | 0.42 | 0.44 |
| AgeP50CI | 1115 | 0.43 | 0.39 | 0.15 | 1.20 | 1.86 | 1.10 | 0.11 | 0.42 | 0.43 |
| AgeP95CI | 1115 | 0.60 | 0.55 | 0.22 | 1.63 | 4.29 | 1.86 | 0.22 | 0.59 | 0.61 |

Pubertal growth estimates. ^1^Age at minimum height velocity of *T*, the total height function, from the QEPS-model. ^2^Age at 5% of *P*, the pubertal growth function, from the QEPS-model. ^3^Age at peak height velocity from the *T*-function. ^4^Age at peak height velocity from the *P-*function. ^5^Age at 50% of the *P-*function. ^6^Age at 95% of the *P-*function. CI = half of the estimated 95% confidence interval in years for the pubertal growth estimates.

**Table A3C The *MathSelect* (MS) function for subgroup selection. MS** **<0.68**

| **Girls** | | | | | | | | | | |
| --- | --- | --- | --- | --- | --- | --- | --- | --- | --- | --- |
| **Variable** | **N** | **Mean** | **Medi-an** | **SD** | **Skew-ness** | **Kurto-sis** | **Max** | **Min** | **Lower 95%** | **Upper 95%** |
|  |  |  |  |  |  |  |  |  | **CL for Mean** | **CL for Mean** |
| AgeTonset^1^ | 753 | 9.20 | 9.17 | 0.94 | 0.097 | -0.041 | 12.23 | 6.37 | 9.13 | 9.27 |
| AgeP5^2^ | 754 | 9.83 | 9.80 | 0.92 | 0.077 | -0.066 | 13.03 | 7.3 | 9.76 | 9.90 |
| AgeTPHV^3^ | 753 | 11.81 | 11.78 | 0.92 | 0.065 | -0.079 | 15.09 | 9.39 | 11.74 | 11.87 |
| AgePPHV^4^ | 754 | 11.98 | 11.96 | 0.91 | 0.078 | -0.082 | 15.23 | 9.51 | 11.91 | 12.04 |
| AgeP50^5^ | 754 | 12.05 | 12.03 | 0.91 | 0.078 | -0.083 | 15.31 | 9.59 | 11.99 | 12.12 |
| AgeP95^6^ | 754 | 14.61 | 14.61 | 0.92 | 0.078 | -0.11 | 17.93 | 12.23 | 14.55 | 14.68 |
| AgeTonsetCI | 754 | 0.72 | 0.71 | 0.18 | 0.36 | -0.17 | 1.31 | 0.22 | 0.71 | 0.74 |
| AgeP5CI | 753 | 0.77 | 0.75 | 0.21 | 0.45 | 0.012 | 1.46 | 0.21 | 0.75 | 0.78 |
| AgeTPHVCI | 754 | 0.52 | 0.50 | 0.15 | 0.54 | 0.15 | 1.08 | 0.14 | 0.50 | 0.53 |
| AgePPHVCI | 754 | 0.48 | 0.47 | 0.14 | 0.37 | -0.27 | 0.88 | 0.14 | 0.47 | 0.49 |
| AgeP50CI | 754 | 0.48 | 0.46 | 0.14 | 0.38 | -0.26 | 0.88 | 0.13 | 0.47 | 0.49 |
| AgeP95CI | 754 | 0.60 | 0.59 | 0.15 | 0.37 | -0.10 | 1.11 | 0.16 | 0.59 | 0.61 |
| **Boys** | | | | | | | | | | |
| **Variable** | **N** | **Mean** | **Medi-an** | **SD** | **Skew-ness** | **Kurto-sis** | **Max** | **Min** | **Lower 95%** | **Upper 95%** |
|  |  |  |  |  |  |  |  |  | **CL for Mean** | **CL for Mean** |
| AgeTonset^1^ | 746 | 10.71 | 10.71 | 0.87 | -0.07 | 0.14 | 13.65 | 7.82 | 10.64 | 10.77 |
| AgeP5^2^ | 746 | 11.75 | 11.77 | 0.87 | -0.09 | 0.06 | 14.60 | 8.82 | 11.69 | 11.81 |
| AgeTPHV^3^ | 746 | 13.63 | 13.64 | 0.87 | -0.07 | 0.02 | 16.37 | 10.69 | 13.56 | 13.69 |
| AgePPHV^4^ | 746 | 13.70 | 13.71 | 0.87 | -0.07 | 0.03 | 16.45 | 10.77 | 13.64 | 13.77 |
| AgeP50^5^ | 746 | 13.77 | 13.78 | 0.87 | -0.07 | 0.02 | 16.51 | 10.83 | 13.70 | 13.83 |
| AgeP95^6^ | 746 | 16.06 | 16.06 | 0.89 | -0.04 | -0.03 | 18.68 | 13.12 | 16.00 | 16.12 |
| AgeTonsetCI | 746 | 0.62 | 0.61 | 0.14 | 0.29 | -0.24 | 1.14 | 0.28 | 0.61 | 0.63 |
| AgeP5CI | 746 | 0.63 | 0.62 | 0.16 | 0.42 | -0.08 | 1.20 | 0.27 | 0.62 | 0.65 |
| AgeTPHVCI | 746 | 0.39 | 0.38 | 0.11 | 0.42 | -0.09 | 0.71 | 0.11 | 0.39 | 0.40 |
| AgePPHVCI | 746 | 0.38 | 0.37 | 0.10 | 0.41 | -0.12 | 0.67 | 0.11 | 0.37 | 0.39 |
| AgeP50CI | 746 | 0.38 | 0.36 | 0.10 | 0.41 | -0.12 | 0.67 | 0.11 | 0.37 | 0.38 |
| AgeP95CI | 746 | 0.53 | 0.51 | 0.13 | 0.68 | 1.00 | 1.19 | 0.23 | 0.52 | 0.54 |

Pubertal growth estimates. ^1^Age at minimum height velocity of *T*, the total height function, from the QEPS-model. ^2^Age at 5% of *P*, the pubertal growth function, from the QEPS-model. ^3^Age at peak height velocity from the *T*-function. ^4^Age at peak height velocity from the *P-*function. ^5^Age at 50% of the *P-*function. ^6^Age at 95% of the *P-*function. CI = half of the estimated 95% confidence interval in years for the pubertal growth estimates.

| **Table A4 CDF distributions with parameter estimates for constructing *MathSelect*** | | | | | |
| --- | --- | --- | --- | --- | --- |
| Input MathSelect variable | CDF distribution | CDF parameters | | | |
|  |  | Girls | | Boys | |
|  |  | mu | sigma | mu | sigma |
| *T_SDerror_*^1^ | loglogistic | -0.056 | 0.15 | -0.0061 | 0.15 |
| *AgeP50_CI_*^2^ | loglogistic | -0.68 | 0.22 | -0.91 | 0.20 |
| *P_heightscaleCI_*^3^ | loglogistic | -1.54 | 0.18 | -1.92 | 0.16 |
| *P_timescaleCI_*^4^ | loglogistic | -1.48 | 0.22 | -1.50 | 0.21 |
| *SP_heightinterceptCI_*^5^ | loglogistic | -1.51 | 0.20 | -1.57 | 0.18 |
| *SP_heightscaleCI_*^6^ | loglogistic | -0.10 | 0.19 | -0.23 | 0.18 |
| *E_timescaleCI_*^7^ | loglogistic | -2.50 | 0.19 | -2.56 | 0.18 |
| *ΔTmaxAH*^8^ | logistic | 0.33 | 0.26 | 0.09 | 0.30 |
| *Penalty*^9^ | normal | -2.16 | 0.48 | -2.11 | 0.51 |
| *MaxCDF*^10^ | Beta | 3.65 | 0.84 | 3.65 | 0.83 |

^1^The standard error of the residuals from the total height function, *T*-function. ^2^Half of the estimated 95% confidence interval for age at which 50% of the pubertal growth *P* is reached.  ^3^Half of 95% CI for the individual height scale ratio, modifying the height scale of the pubertal growth *P*.  ^4^Half of 95% CI for the individual time scale ratio of the *P*-function, modifying the time scale of the pubertal growth. ^5^Half of 95% CI for the individual height intercept parameter in SP-scores, at age 3.25 years for girls and 3.64 years for boys (corrected for gestational age). ^6^Half of 95% CI for the individual height scale parameter in SP-scores.^7^Half of 95% CI for the individual height scale ratio of the *E-*function, modifying the height scale of the exponential growth function *E*. ^8^Maximum modelled total height *T*, minus adult height. ^9^Logit transformed penalty ratio *Logit(PenRatio)* from fitting the *T*-function. ^10^Individual maximum cumulative probability out of nine *MathSelect* step one cumulative distribution functions.
