# Supplementary figures and images for: Insight into human pubertal growth by applying the QEPS growth model
Source: BMC Pediatr. 2017 Apr 19;17:107. doi: 10.1186/s12887-017-0857-1 (PMC5395895; doi:10.1186/s12887-017-0857-1)

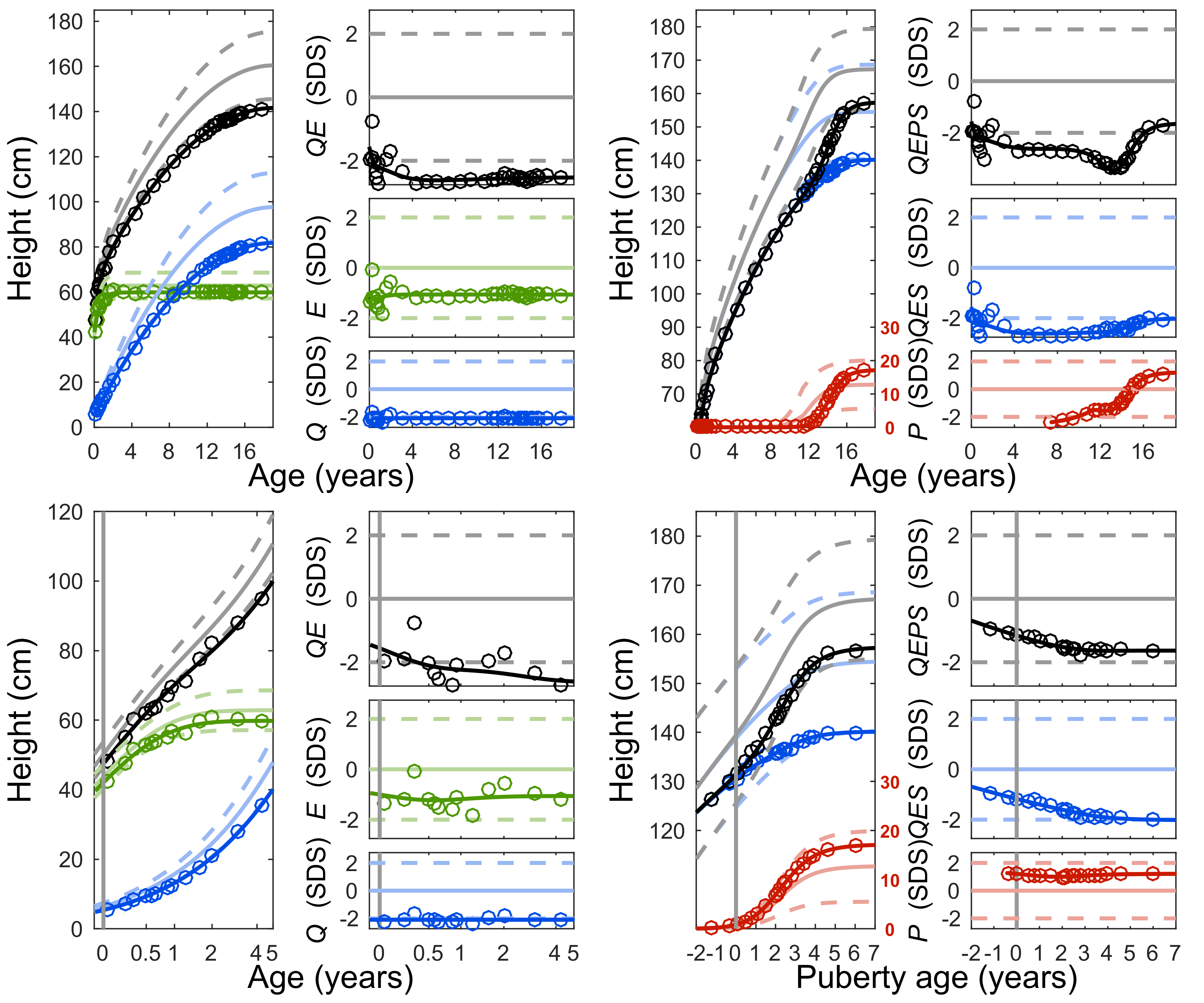

Supplement: Additional file 1: — The first two sections explain pubertal variables of the QEPS-model in more detail in texts, figures and tables for the general pubertal growth, section A.1:1 and the individual variation in pubertal growth, section A.1:2, and the PQ -ratio in A1:3. The construction of the mathematical selection criterion, MathSelect, is described in section A.2:1, in texts, figures and tables, and extreme possible values of the nine input variables corresponding with MathSelect values are computed in section A.2:2. (ZIP 4423 kb) [file 12887_2017_857_MOESM1_ESM.zip › FigA1.A160216R3.png]

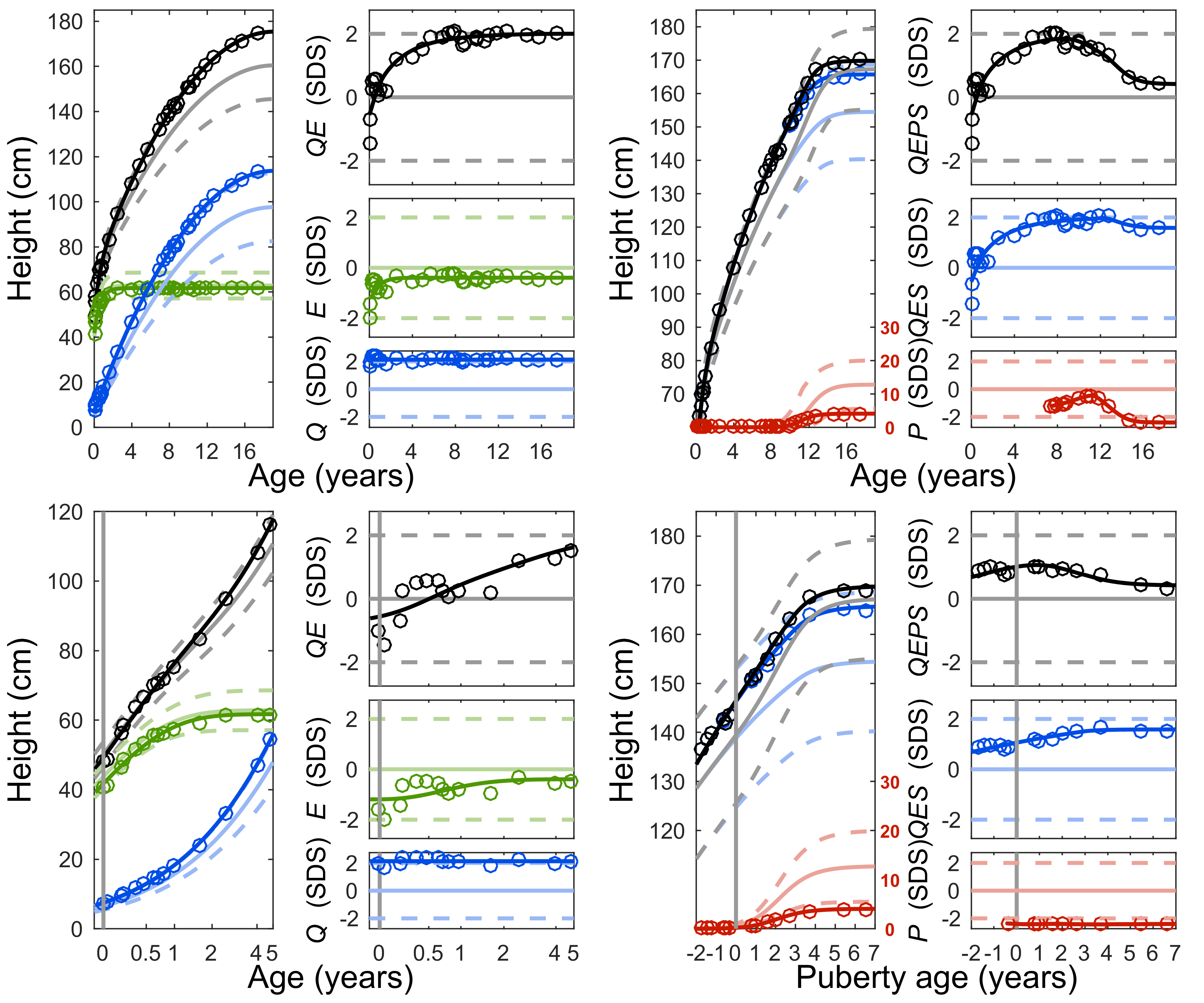

Supplement: Additional file 1: — The first two sections explain pubertal variables of the QEPS-model in more detail in texts, figures and tables for the general pubertal growth, section A.1:1 and the individual variation in pubertal growth, section A.1:2, and the PQ -ratio in A1:3. The construction of the mathematical selection criterion, MathSelect, is described in section A.2:1, in texts, figures and tables, and extreme possible values of the nine input variables corresponding with MathSelect values are computed in section A.2:2. (ZIP 4423 kb) [file 12887_2017_857_MOESM1_ESM.zip › FigA1.B160216R3.png]

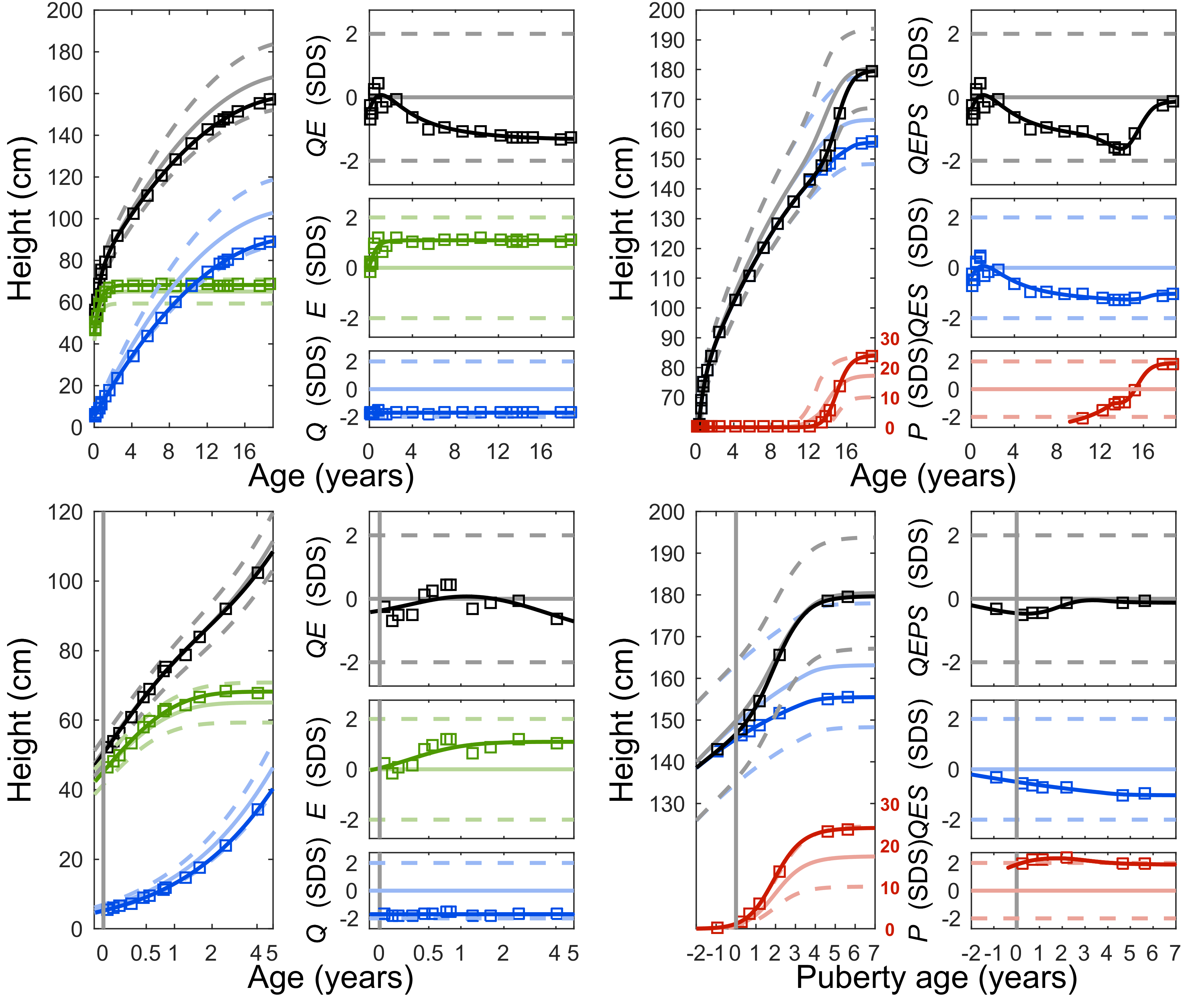

Supplement: Additional file 1: — The first two sections explain pubertal variables of the QEPS-model in more detail in texts, figures and tables for the general pubertal growth, section A.1:1 and the individual variation in pubertal growth, section A.1:2, and the PQ -ratio in A1:3. The construction of the mathematical selection criterion, MathSelect, is described in section A.2:1, in texts, figures and tables, and extreme possible values of the nine input variables corresponding with MathSelect values are computed in section A.2:2. (ZIP 4423 kb) [file 12887_2017_857_MOESM1_ESM.zip › FigA1.C160216R3.png]

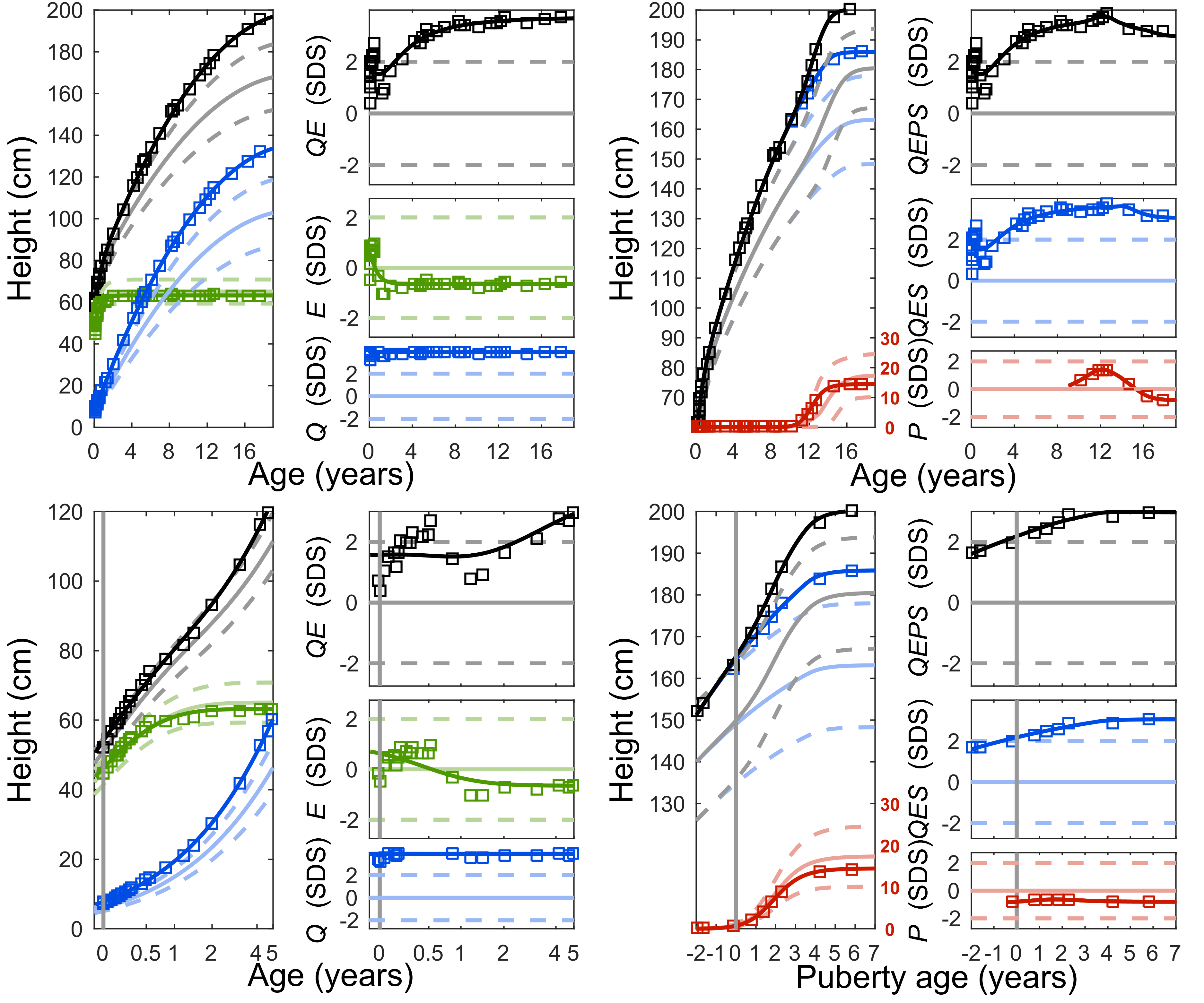

Supplement: Additional file 1: — The first two sections explain pubertal variables of the QEPS-model in more detail in texts, figures and tables for the general pubertal growth, section A.1:1 and the individual variation in pubertal growth, section A.1:2, and the PQ -ratio in A1:3. The construction of the mathematical selection criterion, MathSelect, is described in section A.2:1, in texts, figures and tables, and extreme possible values of the nine input variables corresponding with MathSelect values are computed in section A.2:2. (ZIP 4423 kb) [file 12887_2017_857_MOESM1_ESM.zip › FigA1.D160216R3.png]

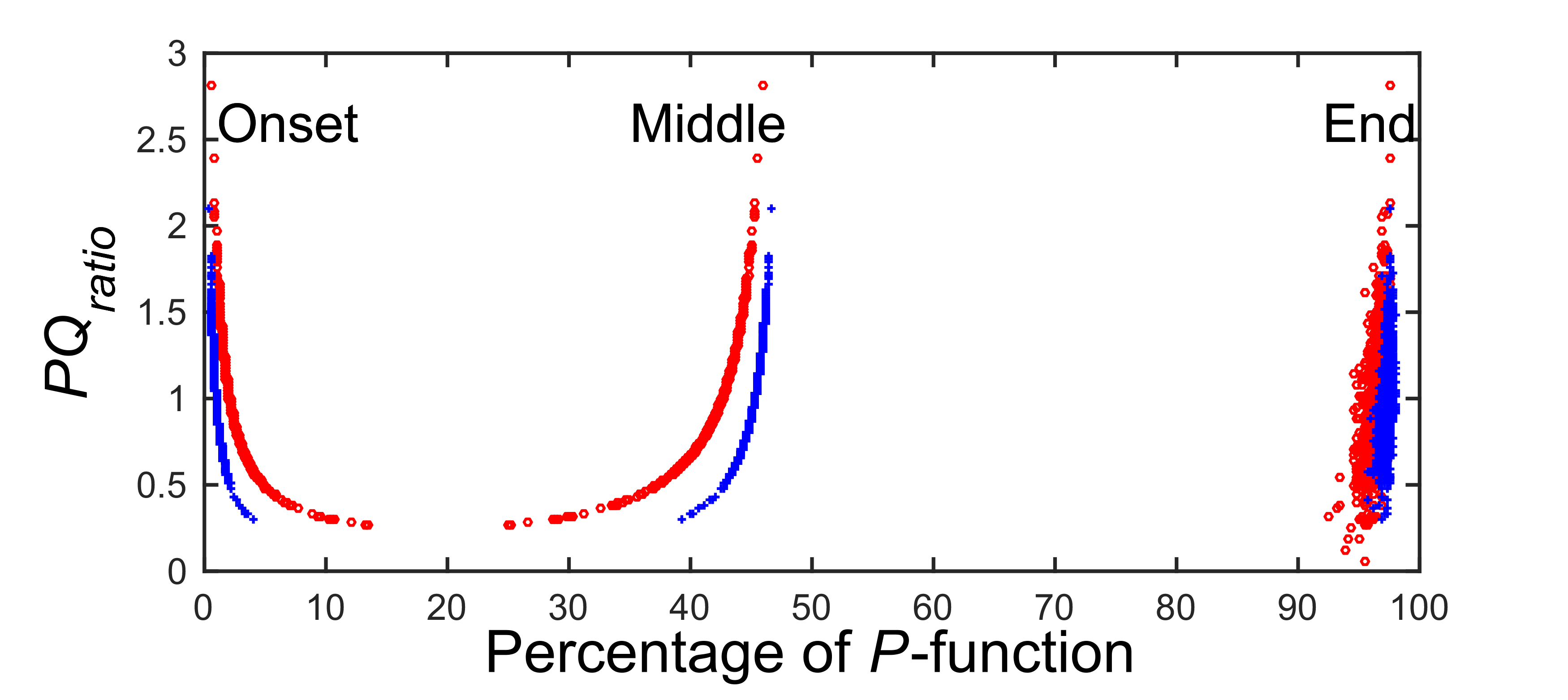

Supplement: Additional file 1: — The first two sections explain pubertal variables of the QEPS-model in more detail in texts, figures and tables for the general pubertal growth, section A.1:1 and the individual variation in pubertal growth, section A.1:2, and the PQ -ratio in A1:3. The construction of the mathematical selection criterion, MathSelect, is described in section A.2:1, in texts, figures and tables, and extreme possible values of the nine input variables corresponding with MathSelect values are computed in section A.2:2. (ZIP 4423 kb) [file 12887_2017_857_MOESM1_ESM.zip › FigA2.160216R3.png]

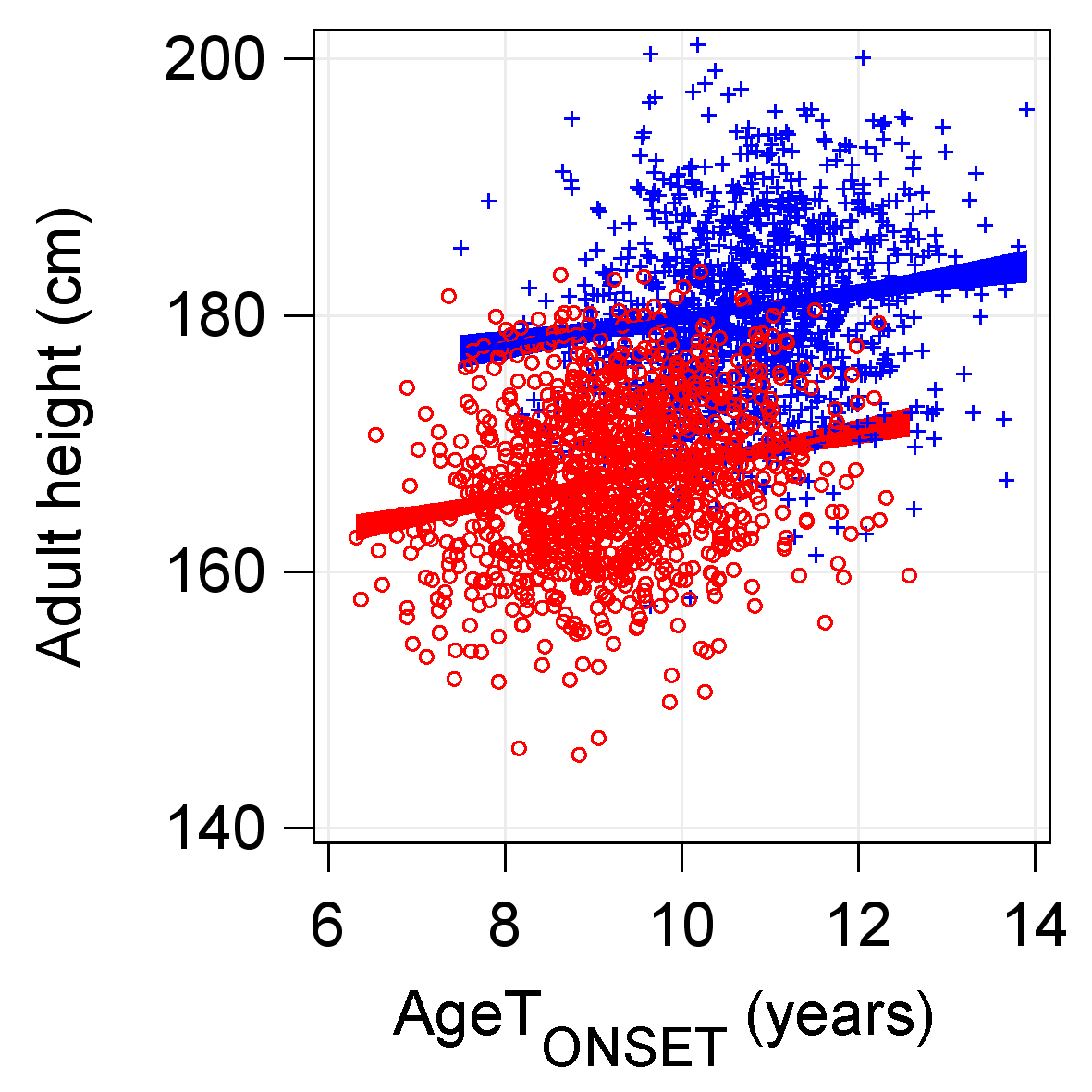

Supplement: Additional file 1: — The first two sections explain pubertal variables of the QEPS-model in more detail in texts, figures and tables for the general pubertal growth, section A.1:1 and the individual variation in pubertal growth, section A.1:2, and the PQ -ratio in A1:3. The construction of the mathematical selection criterion, MathSelect, is described in section A.2:1, in texts, figures and tables, and extreme possible values of the nine input variables corresponding with MathSelect values are computed in section A.2:2. (ZIP 4423 kb) [file 12887_2017_857_MOESM1_ESM.zip › FigA3.160216R3.png]

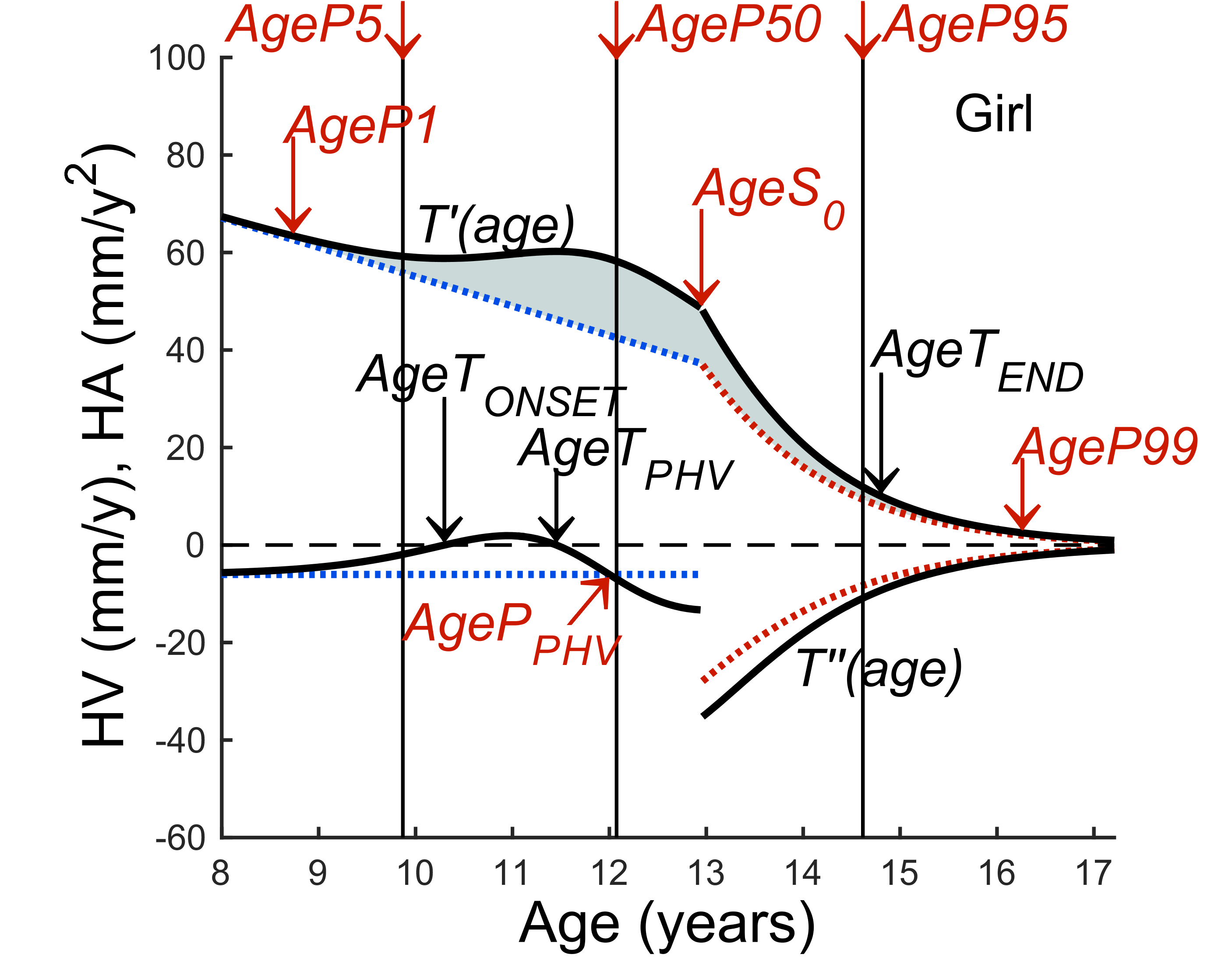

Supplement: Additional file 1: — The first two sections explain pubertal variables of the QEPS-model in more detail in texts, figures and tables for the general pubertal growth, section A.1:1 and the individual variation in pubertal growth, section A.1:2, and the PQ -ratio in A1:3. The construction of the mathematical selection criterion, MathSelect, is described in section A.2:1, in texts, figures and tables, and extreme possible values of the nine input variables corresponding with MathSelect values are computed in section A.2:2. (ZIP 4423 kb) [file 12887_2017_857_MOESM1_ESM.zip › FigA4.160216R3.png]

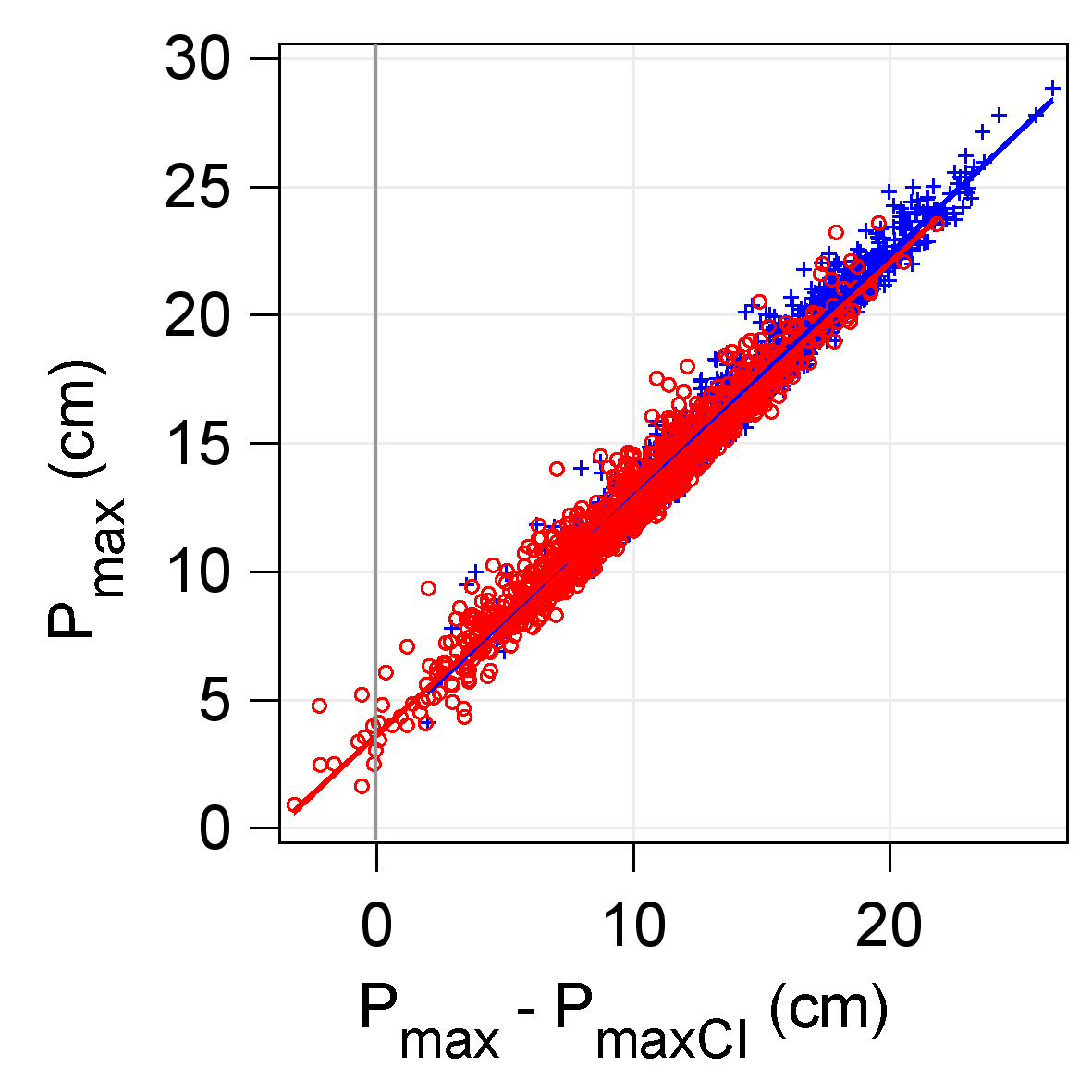

Supplement: Additional file 1: — The first two sections explain pubertal variables of the QEPS-model in more detail in texts, figures and tables for the general pubertal growth, section A.1:1 and the individual variation in pubertal growth, section A.1:2, and the PQ -ratio in A1:3. The construction of the mathematical selection criterion, MathSelect, is described in section A.2:1, in texts, figures and tables, and extreme possible values of the nine input variables corresponding with MathSelect values are computed in section A.2:2. (ZIP 4423 kb) [file 12887_2017_857_MOESM1_ESM.zip › FigA5.160216R3.png]
